# Predicting ICU Delirium with a Regularized Logistic Regression Model: Device-Support Burden as an Informative Predictor Domain in a Turkish Cohort

**DOI:** 10.3390/diagnostics16162554

**Published:** 2026-08-13

**Authors:** Muhammed Sezai Bazna, Fatih Okumuş

**Affiliations:** 1 Department of Intensive Care Unit, Ankara Etlik City Hospital, Ankara 06170, Türkiye; baznacicero@gmail.com; 2 Department of Software Engineering, Inonu University, Malatya 44280, Türkiye

**Keywords:** delirium, intensive care unit, elastic-net logistic regression, Turkish electronic medical records, calibration, TRIPOD

## Abstract

**Background/Objectives:** Delirium is prevalent among intensive care unit (ICU) patients and is associated with prolonged ventilation, longer ICU stays, increased mortality, and post-discharge cognitive impairment. Because most current prediction tools were developed using European cohorts, their transportability to other ICU settings remains unclear. The device support burden has received limited attention, and preprocessing data leakage remains a secondary methodological concern. **Methods:** This prospective study enrolled consecutive adults from the Internal Medicine ICU of Ankara Etlik City Hospital. Fifty predictors from electronic health records were documented at ICU admission or within the first 24 h. Device support variables were assessed as a distinct domain using ablation analysis. We developed an elastic net regularized logistic regression model and validated it using 5 × 4-fold nested cross-validation. Preprocessing was restricted to the training folds, and gradient boosting was used as a comparison method. We also evaluated a fixed exploratory 11-predictor reduced model. **Results:** Delirium was detected in 50.8% of patients. The full regularized model achieved an area under the receiver operating characteristic curve (AUROC) of 0.696 (95% CI: 0.613–0.767). The fixed exploratory reduced model had a higher apparent AUROC of 0.756 (95% CI: 0.684–0.819) than the other models. This apparent advantage was absent when feature selection was repeated within each outer training fold (nested reduced model AUROC 0.700). The full-model calibration slope was 0.741 (95% CI: 0.345–1.265). This confidence interval was wide and included the value of 1.0. The fixed reduced-model slope was 0.235 (95% CI: 0.068–0.886), indicating that the predicted probabilities were too extreme. Ablation analysis showed that device support variables added predictive value to clinical and laboratory variables, whereas environmental variables alone did not significantly discriminate between the two groups. **Conclusions:** In this single-center Turkish ICU cohort, regularized logistic regression showed moderate performance in predicting delirium. Nested feature selection did not reproduce the fixed reduced model’s apparent discrimination advantage. Device support variables are best interpreted as markers of care complexity rather than direct causal factors. External validation and recalibration are required before clinical use. Because delirium onset and device support start times were unavailable, the model estimated delirium risk across the ICU stay from predictors documented at ICU admission or within the first 24 h and was not time-anchored.

## 1. Introduction

Delirium is an acute, fluctuating impairment of attention, awareness, and cognition, with a reported incidence that varies by ICU population and screening method [1,2,3]. Commonly validated ICU delirium assessment tools include the Confusion Assessment Method for the ICU (CAM-ICU) and the Intensive Care Delirium Screening Checklist [4,5]. Studies using validated assessment tools have shown that approximately one-third of critically ill patients develop delirium [2,3]. Among patients receiving mechanical ventilation, the reported incidence increases to 60–80% [2,6,7]. Delirium is more than a transient change in mental status. It is associated with prolonged mechanical ventilation, longer ICU stays, increased mortality risk, and cognitive impairment that may persist after discharge [8,9,10]. In addition to its clinical consequences, delirium increases healthcare resource utilization (HRU). In mechanically ventilated patients admitted to medical ICUs, ICU costs were higher among those who experienced delirium than among those who did not [11].

Delirium prevention in the intensive care unit (ICU) primarily relies on multicomponent non-pharmacological interventions. Common strategies include early mobilization, lighter sedation with regular review, sleep preservation, family involvement, spontaneous awakening and breathing trials when appropriate, and structured care bundles, such as the ABCDEF bundle [12,13,14,15]. Consistent implementation of these interventions in routine practice requires considerable clinical time, staffing, and coordination. Consequently, early identification of patients at increased risk of delirium may help clinicians target preventive measures to those most likely to benefit while making more efficient use of available ICU resources [12,13].

Among the available prediction tools, PRE-DELIRIC is one of the most extensively validated models for estimating the risk of ICU delirium. It was developed by van den Boogaard et al. using data from 1613 Dutch ICU admissions and achieved an AUROC of 0.87 in the development cohort [16]. However, subsequent external validation studies have generally reported lower discrimination, with AUROC values ranging from 0.71 to 0.77 [17,18]. Consistent with these findings, a diagnostic test accuracy meta-analysis of seven studies involving 7941 ICU patients reported a pooled sensitivity of 0.76 (95% CI, 0.60–0.87) and specificity of 0.66 (95% CI, 0.45–0.82) for PRE-DELIRIC [19]. Together, these findings indicate that although PRE-DELIRIC remains an important reference model, its predictive performance may vary across different clinical settings.

Device-support variables are only partly represented in the PRE-DELIRIC and its admission-time counterpart, E-PRE-DELIRIC [20]. Mechanical ventilation is routinely included, whereas other markers of care complexity, such as nasogastric tubes, central venous catheters, vasopressor therapy, high-flow oxygen, and environmental exposures, are incorporated less consistently. In local ICU settings, these variables may provide additional information beyond conventional clinical predictors by reflecting the intensity of organ support, nursing workload, invasive monitoring, respiratory support strategies, nutritional support, and documentation practices.

Simultaneously, recent machine learning studies have reported high AUROC values for delirium prediction; however, these estimates may not accurately represent the performance of truly unseen data [21]. One important reason is preprocessing leakage, which occurs when imputation parameters, encoding schemes, or scaling coefficients are derived from the entire dataset prior to cross-validation. Consequently, information from the test folds can inadvertently influence model development, leading to overly optimistic performance estimates and reduced reproducibility [22].

The transportability of delirium prediction models is particularly relevant in Turkish ICUs. A Turkish validation study of PRE-DELIRIC reported only moderate discrimination in this setting [23] (Section 2.4), highlighting the importance of evaluating prediction models within patient populations, clinical workflows, and electronic health record systems in which they are intended to be used.

In this study, we developed a regularized logistic regression model to predict delirium during ICU stay using a prospective cohort from the Internal Medicine ICU of Ankara Etlik City Hospital. Baseline characteristics were first compared between patients who did and did not develop delirium. Model development incorporated clinical and laboratory variables, device support, and environmental predictors, whereas internal performance was evaluated using a leakage-aware nested cross-validation framework. Given the limited events-per-variable ratio, we explored whether a fixed exploratory reduced model could provide more stable discrimination. Finally, ablation analyses were performed to quantify the individual contributions of the clinical/laboratory, device support, and environmental predictor domains.

Internal validation showed that the full regularized logistic regression model achieved an AUROC of 0.696 (95% CI: 0.613–0.767). Although the fixed exploratory reduced model yielded a higher apparent AUROC (0.756; 95% CI: 0.684–0.819) and lower between-fold variability, this advantage disappeared when feature selection was nested within the cross-validation (AUROC 0.700; Section 4.5). The reduced model also showed poor calibration (calibration slope: 0.235), indicating that probability estimates require recalibration before being used for individual risk predictions. Ablation analyses further demonstrated that device-support variables provided incremental predictive value beyond conventional clinical and laboratory predictors, whereas environmental variables alone did not meaningfully improve the discrimination.

This study makes three main contributions to the literature on ICU delirium prediction:

- This study presents a locally developed and internally validated prediction model based on routinely collected electronic health record data from a tertiary ICU in Türkiye.
- It evaluates device-support variables as a separate predictor domain and quantifies their incremental contributions through ablation analyses.
- It provides a transparent model development framework by combining leakage-aware preprocessing, internal validation, discrimination, calibration, clinical utility assessment, and explicit consideration of a low events-per-variable setting.

Accordingly, this study is intended as a transparent model-development study that provides a foundation for future validation rather than an immediately deployable clinical prediction tool. External validation and recalibration are essential before implementation in routine clinical practice.

## 2. Related Works

### 2.1. Clinical Predictors of ICU Delirium

The clinical determinants of ICU delirium have shown variability across studies, reflecting differences in patient populations, screening approaches, sedation practices, and case mix. Nevertheless, several risk factors have been consistently identified across diverse cohorts. In a systematic review of 33 studies, Zaal et al. identified age, prior cognitive impairment, mechanical ventilation, metabolic acidosis, and higher APACHE-II scores as established risk factors for ICU delirium [24]. Similar findings were reported by Ouimet et al. in a prospective cohort of 820 patients, in which delirium occurred in 31.8% of patients and was associated with older age, previous cognitive impairment, mechanical ventilation, and sedation [25].

Device-related factors are also relevant predictors of ICU delirium but should be interpreted primarily as markers of illness severity and care complexity, rather than direct causal factors. Van Rompaey et al. reported that the presence of tube devices, including nasogastric tubes, urinary catheters, and chest drains, remained independently associated with delirium after multivariable adjustment, with odds ratios ranging from 2.88 to 8.07 [26]. These devices are likely to reflect greater disease severity, the need for invasive treatment, reduced mobility, patient discomfort, and increased nursing and monitoring requirements. Accordingly, in the present study, device support variables were considered indicators of care complexity rather than direct causes of delirium.

Laboratory and nutritional markers may provide complementary information for delirium-risk assessment. Observational studies have reported an association between low serum albumin concentrations and delirium [27,28], possibly because hypoalbuminemia reflects systemic inflammation, malnutrition, acute illness severity, or a combination of these factors.

### 2.2. Structured EHR-Based Machine Learning Prediction Models

Structured electronic health record (EHR) data are increasingly used to develop prediction models for ICU delirium because they capture routinely collected clinical information, including demographic characteristics, laboratory results, vital signs, illness severity scores, and treatment-related variables of patients. However, despite these advantages, models developed from structured EHR data often show reduced performance when applied to different hospitals, databases, or time periods, highlighting the challenges of model transportability and generalizability.

This pattern has been illustrated by several recent EHR-based prediction models. The PRIDE algorithm achieved excellent internal discrimination (AUROC, 0.916); however, its performance declined to an AUROC of 0.721 during external validation [21]. Similarly, Zhang et al. developed an XGBoost model for sepsis-associated delirium using the MIMIC-IV database and externally validated it with the eICU-CRD database, reporting AUROC values of 0.793 and 0.701 for the internal and external validation cohorts, respectively [29]. Wang et al. developed and externally validated admission-based models for early delirium prediction, further emphasizing the importance of evaluating model performance beyond the development cohort [30].

Together, these studies demonstrate that structured EHR-based models can capture clinically relevant signals, but maintaining their performance across different clinical settings remains a challenge. Differences in documentation practices, variable definitions, patterns of missing data, sedation strategies, and delirium screening protocols may influence model transportability. Systematic reviews have reached similar conclusions in the past. Ruppert et al. identified 23 ICU delirium prediction models, of which only five had undergone external validation in an independent study [31]. Likewise, a recent systematic review and critical appraisal reported a substantial risk of bias across ICU delirium prediction models, particularly in the analysis domain [32]. More broadly, these challenges extend beyond the prediction of delirium. In clinical prediction modelling, preprocessing steps such as imputation, scaling, and encoding can produce overly optimistic performance estimates when applied before, rather than within, cross-validation training folds [22].

### 2.3. Sample Size, Events per Variable, and Model Stability

When the number of candidate predictors is large relative to the number of outcome events, the limited sample size becomes a major challenge for clinical prediction modelling. Under these conditions, regression coefficients may become unstable, confidence intervals may widen, and model performance may appear overly optimistic when evaluated using new data. The concept of events per variable (EPV) was introduced to quantify this issue. Peduzzi et al. demonstrated that logistic regression estimates become increasingly unreliable when the EPV falls below approximately 10 [33]. Although this threshold should not be regarded as an absolute rule, it remains a useful indicator of model stability. Riley et al. subsequently proposed a more comprehensive sample size framework that incorporates the expected model performance, outcome prevalence, and overfitting risk [34]. Despite their methodological differences, both approaches emphasize the same underlying principle: predictive performance becomes less reliable as the amount of information available per predictor decreases. Penalized regression can reduce overfitting through coefficient shrinkage but cannot compensate for insufficient sample data. Accordingly, the limited information-to-parameter ratio was treated as an inherent constraint in this study. Both the full penalized model and a predefined exploratory reduced model were evaluated within this context.

### 2.4. Methodological Gaps Addressed by the Study

Current evidence indicates that the performance of ICU delirium prediction models strongly depends on the population and methodology used for model development. External validation remains relatively uncommon, and systematic reviews have shown that only a minority of published models have been evaluated beyond their original development settings [31,32]. Calibration represents another recurring limitation of this literature. Although most studies emphasize discrimination, an accurate agreement between the predicted and observed risks is equally important if prediction models are intended to support individual patient care [32].

Methodological rigor in model development is another important consideration. Structured EHR data typically require preprocessing steps, such as imputation, encoding, and scaling, before model fitting. When these procedures are performed before, rather than within, cross-validation, information from the test folds can inadvertently influence model development, leading to overly optimistic performance estimates [21,22]. This concern becomes particularly important in low-information settings, where a limited sample size and a large number of candidate predictors increase the risk of unstable coefficient estimates and overfitting [33,34].

Another limitation concerns the representation of device-support variables. Although PRE-DELIRIC and E-PRE-DELIRIC include mechanical ventilation, they capture other indicators of care complexity, such as nasogastric tubes, central venous catheters, vasopressor therapy, and high-flow oxygen, only to a limited extent [16,20]. Previous clinical studies suggest that these variables are associated with delirium; however, they are more appropriately interpreted as markers of illness severity and care complexity than as direct causal factors [26]. Moreover, their predictive value may vary across ICUs because of differences in clinical practice, staffing, and the patterns of documentation.

Model transportability is particularly relevant in Turkish ICUs. In a Turkish validation study involving 172 ICU patients, PRE-DELIRIC achieved only moderate discrimination (AUROC, 0.649; 95% CI, 0.538–0.760) [23]. Rather than suggesting that locally developed models are inherently superior, these findings underscore the importance of evaluating prediction models within the patient populations, clinical settings, and electronic health record environments in which they are intended to be used.

Beyond discrimination, calibration and clinical utility are essential considerations for prediction models intended to support patient-level decision-making. Although the AUROC remains the most frequently reported performance metric, it does not indicate whether a model provides a meaningful clinical benefit. Decision curve analysis addresses this aspect but has been reported inconsistently in previous ICU delirium prediction studies [30].

Accordingly, the present study was designed as a transparent model development investigation with internal validation rather than as a clinically deployable prediction tool. Model development incorporated leakage-aware preprocessing, joint assessment of discrimination and calibration, decision curve analysis, explicit consideration of the low events-per-variable setting, and separate evaluation of device support variables as an independent predictor domain. External validation and recalibration are necessary before routine clinical implementation.

## 3. Materials and Methods

### 3.1. Study Setting and Ethical Approval

This prospective single-center study was conducted in the Internal Medicine Intensive Care Unit (ICU) of Ankara Etlik City Hospital (Ankara, Türkiye). The study was conducted prospectively between 6 November 2024 and 5 November 2025, corresponding to the one-year period authorized by the ethics committee. The hospital is a major tertiary public referral center serving both the metropolitan Ankara area and Central Anatolia region. The Internal Medicine ICU is a 20-bed closed unit staffed by intensivists, resident physicians, and critical-care nurses. Routine delirium prevention practices included daily sedation assessment, spontaneous awakening and breathing trials when clinically appropriate, early mobilization, sleep promotion and family involvement. These measures were implemented as part of the standard clinical care and were not introduced specifically for this study. The study protocol was approved by the Ankara Etlik City Hospital Scientific Research Evaluation and Ethics Committee (decision no. AEŞH-BADEK-2024-961; 6 November 2024) and was performed in accordance with the Declaration of Helsinki. Written informed consent was obtained from patients with decision-making capacity. When a patient lacked decision-making capacity, consent was obtained from a legally authorized representative before inclusion. Before the analysis, all directly identifiable information, including patient names and Turkish national identity numbers, was removed from the dataset.

For prediction modelling, clinical and device-support variables were recorded at ICU admission or within the first 24 h of ICU stay using routinely documented physician and nursing records. Environmental exposure was recorded as a binary variable using a structured checklist. Because the dataset did not retain timestamps for these observations, a more precise temporal reference for environmental variables could not be established.

### 3.2. Participants and Outcome Definition

During the approved study period, 399 ICU admissions to the Internal Medicine ICU were recorded and evaluated against the study eligibility criteria, and one index admission per patient was retained for analysis, giving a final cohort of 193 patients. For seven patients with more than one ICU admission during the study period, only the second admission was retained as the index admission for analysis. Admission records not retained for analysis included non-eligible admissions and non-index admissions from patients with more than one ICU stay. Patients were included if they were aged ≥18 years, had an ICU stay of at least 24 h, had no documented history of dementia, active psychosis, or severe intellectual disability at admission, and had at least one Richmond Agitation–Sedation Scale (RASS) score of −2 or higher within the first 72 h of ICU admission. This criterion was applied because delirium assessment using the Confusion Assessment Method for the ICU (CAM-ICU) is not considered valid in patients with deep sedation. Accordingly, patients whose RASS scores remained between −3 and −5 throughout their ICU stay were excluded [4].

All candidate predictors were collected at ICU admission or within the first 24 h of ICU stay, according to the study protocol. However, the dataset did not contain the time of the first CAM-ICU-positive assessment or the start times of the individual device-support interventions. Consequently, a delirium-free landmark at 24 h could not be established, and landmark-based sensitivity analyses were not possible. Therefore, the model estimates the risk of delirium occurring at any time during the ICU stay using predictors collected at ICU admission or within the first 24 h. It should not be interpreted as a time-anchored prediction model, and for patients who developed delirium very early, the temporal sequence between predictor measurements and delirium onset could not be confirmed.

Patients who remained too deeply sedated or unresponsive for a valid delirium assessment were excluded, in accordance with the RASS and CAM-ICU eligibility criteria described above. A previous cerebrovascular event or other central nervous system disease was not an automatic exclusion criterion. Patients with residual motor deficits alone remained eligible whenever cognitive and delirium assessment was feasible.

Delirium was assessed twice daily during morning and evening physician rounds using a structured delirium screening checklist and the Confusion Assessment Method for the ICU (CAM-ICU). The CAM-ICU has demonstrated high diagnostic accuracy when used by trained assessors, with a reported sensitivity of 93–100% and specificity of 98–100% in the original validation study [4], and pooled estimates of 75.5% sensitivity and 95.8% specificity in a meta-analysis of 34 studies [35].

In the present study, the CAM-ICU was administered by the attending intensivist during routine morning and evening ICU rounds. During daytime care and overnight shifts, ICU residents and nurses reported suspected acute changes in mental status, attention, or awareness. These patients were reassessed by the intensivist, who administered or repeated the CAM-ICU as appropriate, made the final clinical determination, and initiated treatment when indicated.

No paired independent assessments were performed; therefore, inter-rater agreement could not be estimated. Reports from residents and nurses served as clinical alerts and did not constitute independent CAM-ICU assessments. As delirium screening was integrated into routine clinical care, assessors were not blinded to the patients’ clinical information.

The primary outcome was the occurrence of at least one CAM-ICU-positive assessment during the ICU stay period. Delirium status was recorded as a single binary variable (1 = at least one CAM-ICU-positive assessment at any time during the ICU stay, 0 = none); patients were not classified at admission, and the timing of the first positive assessment was not recorded in the dataset. Delirium subtype was documented for routine clinical care but was excluded from model development because it becomes known only after delirium has developed and is therefore unavailable at the time of risk prediction.

### 3.3. Predictor Variables and Turkish EMR Parsing

Fifty candidate predictors were extracted from the electronic health records at ICU admission or within the first 24 h of ICU stay. These variables included demographic and clinical characteristics, illness severity scores, laboratory results, device support status, and environmental exposure. Table 1 lists the predictors, measurement types, and coding used in this study. The admission diagnosis variable was highly granular. Before model development, semantically equivalent diagnosis labels that had been recorded using different conventions were harmonized into a single canonical category; this normalization was applied to the raw values before one-hot encoding. Composite diagnoses were not decomposed, and categories that were not clinically equivalent, or whose equivalence could not be established, were kept separate. After harmonization the variable comprised 43 categories, 25 of which were represented by a single patient (Supplementary Table S2). To preserve a leakage-free development process, this variable was one-hot encoded using an encoder fitted separately within each training fold (handle_unknown = “ignore”). No rare category collapse was performed. Consequently, one-hot encoding increased the effective number of model parameters to approximately 92, reducing the effective events-per-parameter ratio well below the nominal EPV of 1.96.

In the hospital information system, some laboratory results were stored as composite text fields rather than individual numeric variables. These fields were parsed into separate laboratory measurements using predefined regular expression rules. Before parsing, non-standard separators were standardized, and values that could not be converted to a numeric format were treated as missing. Parsing failures were uncommon and affected fewer than 2.1% of the laboratory values. This preprocessing step was performed solely to construct an analytical dataset from raw electronic health record data. No outcome information or distributional characteristics from the complete dataset were used in this process.

During model development, all preprocessing steps, including imputation, encoding, and scaling, were performed exclusively within the training fold. To minimize temporal leakage, variables that were unavailable at the intended time of prediction or determined only after delirium onset were excluded from all predictor sets. These variables included delirium subtype, ICU length of stay category, post-diagnosis pharmacological treatment, hospital outcome, and admission date. Delirium subtype was excluded because it was assigned only after the development of delirium. ICU length of stay and hospital outcomes become available only after hospitalization has progressed, whereas pharmacological treatment may represent a consequence rather than a predictor of delirium. The admission date was omitted because it could inadvertently capture temporal trends in clinical practice or documentation during the study period.

### 3.4. Cohort Description and Group Comparisons

Baseline characteristics were compared between patients who did and did not develop delirium. Fisher’s exact test was used for binary variables, the Mann–Whitney U test for continuous and ordinal variables, and Pearson’s χ^2^ test for the multicategory admission diagnosis variable. Because 50 candidate predictors were evaluated, the *p*-values were adjusted using the Benjamini–Hochberg false discovery rate (FDR) procedure. Variables with an FDR-adjusted *q*-value of <0.05 were considered statistically significant. Both unadjusted *p*-values and FDR-adjusted *q*-values are reported in Table 2.

### 3.5. Events per Variable Analysis

Before model development, the relationship between the number of outcome events and candidate predictors was evaluated because limited outcome information could compromise the stability of the regression models. Peduzzi et al. demonstrated in simulation studies that logistic regression estimates become increasingly unreliable when the number of events per variable falls below approximately 10 [33]. Recently, Riley et al. proposed a broader sample-size framework for prediction modelling that incorporates the expected model performance, outcome prevalence, and overfitting risk [34]. Although these approaches differ methodologically, both emphasize the importance of sufficient outcome information for developing reliable and accurate prediction models.

In the study cohort, 98 patients developed delirium, and 50 candidate predictors were available for the model development. The nominal events per variable (EPV) was 1.96 and decreased slightly to 1.90 when calculated using a smaller outcome class of 95 non-events. Because one-hot encoding of the admission diagnosis variable substantially increased the number of model parameters, the effective information-per-parameter ratio was lower than the nominal EPV values. Therefore, low EPV was considered the principal limitation of the full model, and penalized logistic regression was used to reduce coefficient instability. In addition, a prespecified exploratory reduced model comprising 11 predictors identified in an earlier pure-ridge bootstrap analysis was evaluated. Although this increased the nominal EPV to 8.6, its apparent performance should be interpreted cautiously because the predictor set was derived from the same development dataset.

### 3.6. Model Development and Internal Validation Pipeline

**Figure 1:**
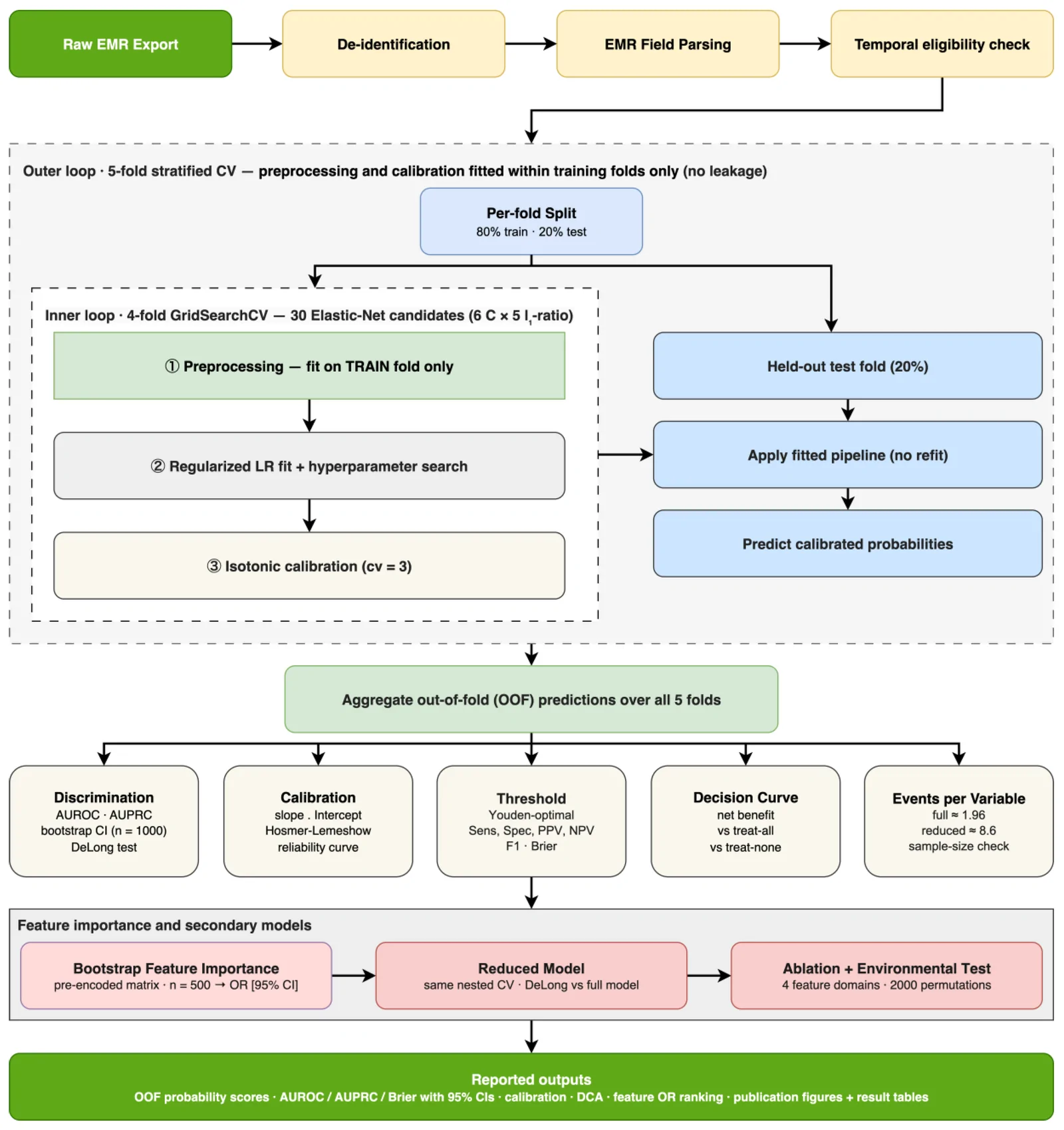
Nested cross-validation workflow used for ICU delirium prediction. The dashed boundary denotes an outer cross-validation loop. Within each outer training fold, preprocessing steps, including imputation, one-hot encoding, and standardization, were fitted exclusively to the training data and then applied unchanged to the corresponding held-out outer fold. Hyperparameters were optimized within an inner four-fold cross-validation loop by evaluating 30 elastic net parameter combinations, followed by isotonic probability calibration. Out-of-fold (OOF) predictions from the outer folds were aggregated to evaluate the discrimination, calibration, threshold-based performance, and decision curve analysis. The prespecified exploratory reduced model was evaluated using the same nested workflow, whereas the nested reduced model repeated the earlier pure-ridge bootstrap variable selection procedure independently within each outer training fold. Because the reduced predictor set was derived from the development dataset, this comparison should be interpreted as an internal, exploratory analysis. OOF, out-of-fold; AUROC, area under the receiver operating characteristic curve; AUPRC, area under the precision–recall curve; DCA, decision curve analysis; OR, odds ratio; CI, confidence interval.

Figure 1 summarizes the model development and internal validation workflow. All analyses were performed in Python 3.12.10 using scikit-learn 1.8.0, pandas 3.0.2, NumPy 2.2.6, SciPy 1.17.1, and statsmodels 0.14.6, with the random seed fixed at 42 for every stochastic step, including the cross-validation splits, the bootstrap resampling, and the permutation test. The modeling strategy used a stratified 5-fold outer cross-validation loop with a stratified 4-fold inner loop for hyperparameter selection; both loops shuffled the data before splitting, under the fixed seed. Within each outer training fold, preprocessing, regularized logistic regression tuning, and isotonic calibration were sequentially performed. Preprocessing included imputation, one-hot encoding, and standardization, and these steps were fitted only to the training portion of each fold. The calibrated model was then applied to the corresponding held-out outer fold without re-fitting, producing an out-of-fold predicted probability for each patient. The same out-of-fold predictions were used for discrimination, calibration, threshold-based metrics, and decision curve analyses. The imputation parameters, encoder vocabularies, scaling parameters, model coefficients, and calibration mappings were estimated without using information from the held-out outer folds. Within each outer training fold, an inner 4-fold grid search selected C and l1_ratio from 30 parameter combinations using the AUROC as the optimization criterion. The selected pipeline was subsequently calibrated within the outer training fold using CalibratedClassifierCV (method = “isotonic”, cv = 3), which for a classification target splits that training fold into three stratified folds. The held-out outer fold was not used for preprocessing, hyperparameter tuning, model fitting or calibration. All logistic regression models were fitted with class_weight = “balanced”. Because the outcome was nearly balanced (98 delirium vs. 95 non-delirium cases), no additional resampling was conducted.

### 3.7. Preprocessing

Preprocessing was embedded within the nested cross-validation workflow and performed separately for each outer training fold. Continuous and ordinal variables were imputed using the median and subsequently standardized to zero mean and unit variance. Median imputation was chosen because several laboratory variables showed skewed distributions and occasionally contained extreme values, particularly creatinine and blood urea nitrogen levels in patients with acute kidney injury. The admission diagnosis was treated as a categorical variable, with missing values imputed using the most frequent category within the training fold before one-hot encoding. Categories observed only in the corresponding held-out fold were ignored during encoding (handle_unknown = “ignore”). Accordingly, all preprocessing parameters, including imputation values, encoder vocabularies, and scaling parameters, were estimated exclusively from the training data and applied unchanged to the held-out fold. Feature-level missingness was low overall, reaching a maximum of 2.07% for platelet count and remaining below 1% for most of the variables. A complete summary of the missing data for each predictor is provided in Supplementary Table S1 (the 50 candidate predictors).

### 3.8. Primary and Comparator Models

Elastic net–penalized logistic regression was selected as the primary prediction model because of the relatively small sample size and large number of candidate predictors [36]. The ridge component stabilizes the coefficient estimates through shrinkage, whereas the lasso component suppresses weak predictors by shrinking some coefficients toward zero. Together, these penalties provide a regularization strategy that is well-suited to low-information settings. Hyperparameters were optimized within the inner four-fold cross-validation loop by maximizing the AUROC across 30 candidate parameter combinations. The most frequently selected configuration across the five outer folds was l1_ratio = 0.5 and C = 0.05, indicating a relatively strong regularization. Following hyperparameter selection, probability calibration was performed using isotonic regression (CalibratedClassifierCV, method = “isotonic”, cv = 3) within each outer training fold before evaluation of the corresponding held-out outer fold.

Gradient boosting was used as a nonlinear comparator to assess whether a more flexible learning algorithm could extract additional predictive information from the same structured predictor set. The hyperparameters were optimized within an identical nested cross-validation framework by tuning the number of trees, tree depth, learning rate, and subsampling ratio. The primary analysis evaluated a predefined 24-point hyperparameter grid, whereas the sensitivity analysis used an expanded 72-point grid. Both analyses followed the same preprocessing and internal validation workflows as the primary model. The comparator was implemented using scikit-learn’s GradientBoostingClassifier.

An untuned logistic regression model was evaluated as a baseline reference. This model used the default scikit-learn settings (L2 penalty, C = 1.0) without hyperparameter optimization or isotonic calibration and was evaluated using the same nested cross-validation framework. The baseline model achieved an AUROC of 0.627 (95% CI, 0.544–0.710), compared with 0.696 for the tuned and calibrated elastic-net model. Because the baseline differed from the primary model in terms of regularization, calibration, and hyperparameter optimization, this comparison should be interpreted as an overall reference rather than as evidence for the isolated effect of hyperparameter tuning.

### 3.9. Reduced Model

Because the full model was developed under low EPV conditions, a fixed reduced model was evaluated to examine whether a smaller predictor set could provide more stable internal performance estimates. The reduced model included 11 predictors identified in an earlier bootstrap analysis under pure-ridge regularization (l1_ratio = 0), for which all bootstrap confidence intervals excluded an odds ratio of 1.0. When these predictors were re-estimated under the elastic-net penalty used in the primary analysis, only vasopressor use and nasogastric tube retained confidence intervals excluding an odds ratio of 1.0. The remaining predictors were retained from the earlier pure-ridge bootstrap analysis, and their current elastic-net coefficient estimates should therefore be regarded as exploratory.

The reduced model was evaluated using the same nested cross-validation framework as the full model, including identical preprocessing, hyperparameter optimization, and isotonic calibration procedures. Reducing the number of predictors increased the nominal EPV to 8.6, although this remained below the conventional target of 10 and did not eliminate concerns regarding model stability.

Because the reduced predictor set was derived from the same development dataset used for model evaluation, any apparent performance advantage may reflect optimism and should be interpreted as an internal exploratory comparison that requires confirmation in an independent external cohort. The AUROC difference between the full and reduced models was assessed using the DeLong test. To quantify the optimism introduced by feature selection, we performed a nested feature selection sensitivity analysis in which the complete bootstrap variable selection procedure was repeated independently within each outer training fold before evaluation on the corresponding held-out fold. Throughout this manuscript, the fixed reduced model refers to the 11-predictor model derived once from the full dataset, whereas the nested reduced model refers to the model in which the same selection procedure was repeated independently within each outer training fold.

### 3.10. Performance Measures and Statistical Testing

The model performance was estimated using aggregated out-of-fold predictions. Discrimination was assessed using the AUROC as the primary measure, and the AUPRC was reported as a complementary measure of performance. The Brier score was used to summarize the accuracy of the predicted probabilities. Calibration was evaluated using the calibration slope, Hosmer–Lemeshow test [37], and reliability plot. Threshold-dependent performance was reported at the Youden-optimal threshold and included sensitivity, specificity, positive predictive value, negative predictive value, F1 score, and accuracy. Confidence intervals for the performance measures were estimated using percentile bootstrap resampling with 1000 iterations [38]. Paired AUROC comparisons between the models were performed using DeLong’s test [39]. The clinical utility was examined using decision curve analysis, which compares the net benefit of model-guided decisions with treat-all and treat-none strategies across clinically relevant threshold probabilities [40]. Threshold probabilities ranging from 0.05 to 0.75 were examined. All statistical tests were two-sided, and statistical significance was set at *p* < 0.05.

### 3.11. Feature Importance Estimation

Feature importance was examined using odds ratios derived from a regularized logistic regression model. Odds ratios (ORs) and their 95% bootstrap confidence intervals (CIs) were estimated using 500 bootstrap resamples. To ensure a consistent feature matrix across the bootstrap samples, the one-hot encoding structure was fixed before resampling. This prevented changes in the indicator columns for admission diagnosis that could otherwise arise when categorical variables with multiple levels were re-sampled.

For the fixed reduced model, bootstrap confidence intervals were re-estimated under the elastic-net specification used in the primary analysis. For the nested reduced model, the earlier pure-ridge bootstrap variable selection procedure was repeated independently within each outer training fold before the fold-specific elastic-net model was tuned, calibrated, and evaluated on the corresponding held-out outer fold.

### 3.12. Ablation Analysis and Environmental Permutation Test

Ablation analyses were performed to examine the contribution of different predictor domains. The nested cross-validation workflow was repeated for four feature sets: clinical/laboratory variables only, environmental variables only, clinical/laboratory plus device-support variables, and all candidate predictors. The Clinical/Laboratory subset comprised demographic, clinical, illness-severity, and laboratory variables, which were fitted together as one modelling group; the reporting domains listed in Table 1 are finer than these three feature sets and were not tested as separate models. AUROC, AUPRC, and Brier scores were reported for each feature set using the bootstrap confidence intervals. Because the environmental-only model produced an AUROC below 0.50, a one-sided permutation test was used to assess whether this result differed from that of the chance. The out-of-fold predicted probabilities from the environmental model were kept fixed, whereas the outcome labels were randomly permuted 2000 times. The *p*-value was calculated as the proportion of permuted AUROCs less than or equal to the observed AUROC. A non-significant result was interpreted as indicating that the below-chance estimate was compatible with random variation rather than a reproducible inverse association.

### 3.13. Reporting Standards

This study was reported in accordance with the TRIPOD statement for multivariable prediction models [41] and TRIPOD+AI guidance for machine learning prediction studies [42].

## 4. Results

### 4.1. Cohort Characteristics

Among the 193 patients who met the eligibility criteria and were included in the analysis, one record per patient, 98 (50.8%) developed delirium during their ICU stay. Fifteen candidate predictors differed between patients with and without delirium in unadjusted analyses (*p* < 0.05), and 11 remained significant after Benjamini–Hochberg false discovery rate correction (*q* < 0.05; Table 2). Four predictors reached raw significance but did not survive correction: age (*q* = 0.052), sex (*q* = 0.080), blood urea nitrogen (*q* = 0.080), and comorbidity count (*q* = 0.128). The predictors that remained significant after correction mainly reflected the illness severity, device support burden, and systemic inflammation. These included vasopressor use, nasogastric tube, SOFA score, noninvasive mechanical ventilation, high-flow oxygen, prior hospitalization, FiO_2_ > 40%, mechanical ventilation, APACHE II score, CRP, and albumin. Patients who developed delirium had higher SOFA scores (median 6 vs. 4), APACHE II scores (22 vs. 18), C-reactive protein levels (95 vs. 54 mg/L), and lower albumin levels (28.5 vs. 32.0 g/L) than those who did not develop delirium.

### 4.2. Events per Variable

The full model was developed under limited events per variable (EPV) conditions. In the analytic cohort, 98 patients developed delirium, and 50 candidate predictors were considered, corresponding to a nominal EPV of 1.96. Using the smaller outcome class of 95 non-events yielded a nominal EPV of 1.90. The effective information-to-parameter ratio was lower than the nominal values because the admission diagnosis variable was expanded through one-hot encoding. Although penalized logistic regression mitigates coefficient instability through shrinkage, it cannot fully compensate for the limited outcome information. Given the observed prevalence of delirium, achieving the conventional EPV target of approximately 10 would require a sample size of approximately 985 patients [33,34]. Accordingly, the full model should be regarded as an internally validated developmental model, rather than a definitive clinical prediction tool. Therefore, a reduced-model analysis was conducted as an exploratory assessment to determine whether a smaller predictor set could provide more stable internal performance estimates. Because this predictor set was derived from the same development dataset, any apparent performance advantage should be interpreted with caution.

### 4.3. Full-Feature Model Performance

The full regularized logistic regression model achieved an AUROC of 0.696 (95% CI, 0.613–0.767), an AUPRC of 0.689, and a Brier score of 0.228 in nested cross-validation. The calibration slope was 0.741, indicating that the predicted probabilities were somewhat extreme. The Hosmer–Lemeshow test was not significant (*p* = 0.109); however, given the limited sample size, this finding should not be interpreted as evidence of good calibration because the test has a low power to detect miscalibration. The AUROC values ranged from 0.588 to 0.839 across the five outer folds, likely reflecting the limited information-to-parameter ratio of the full model. The detailed performance metrics are presented in Table 3.

**Figure 2:**
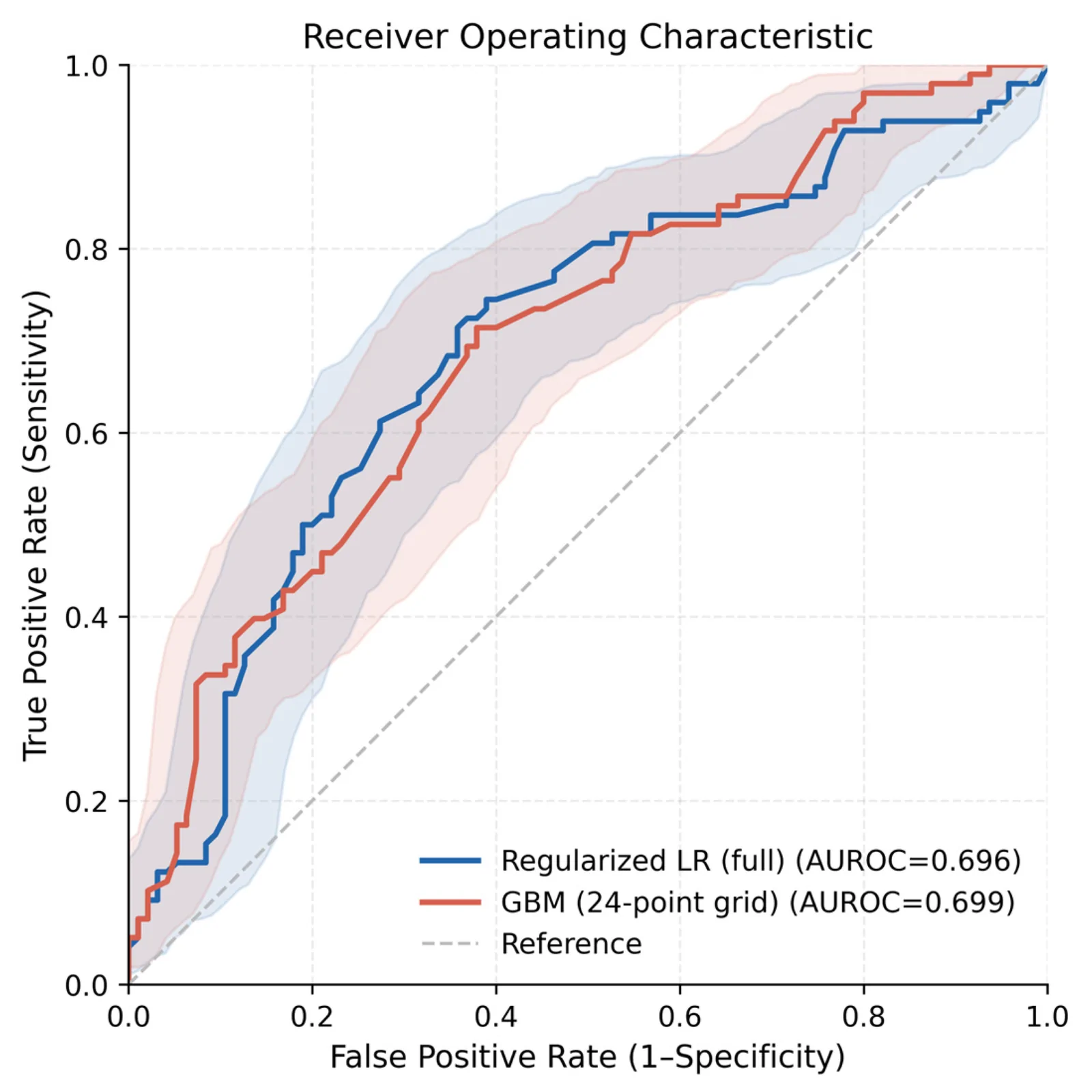
Receiver Operating Characteristic curves with 95% bootstrap confidence bands for regularized LR (full feature, AUROC = 0.696) and the GBM comparator (24-point grid, AUROC = 0.699). Diagonal dashed line = no discrimination reference. DeLong *p* = 0.924.

**Figure 3:**
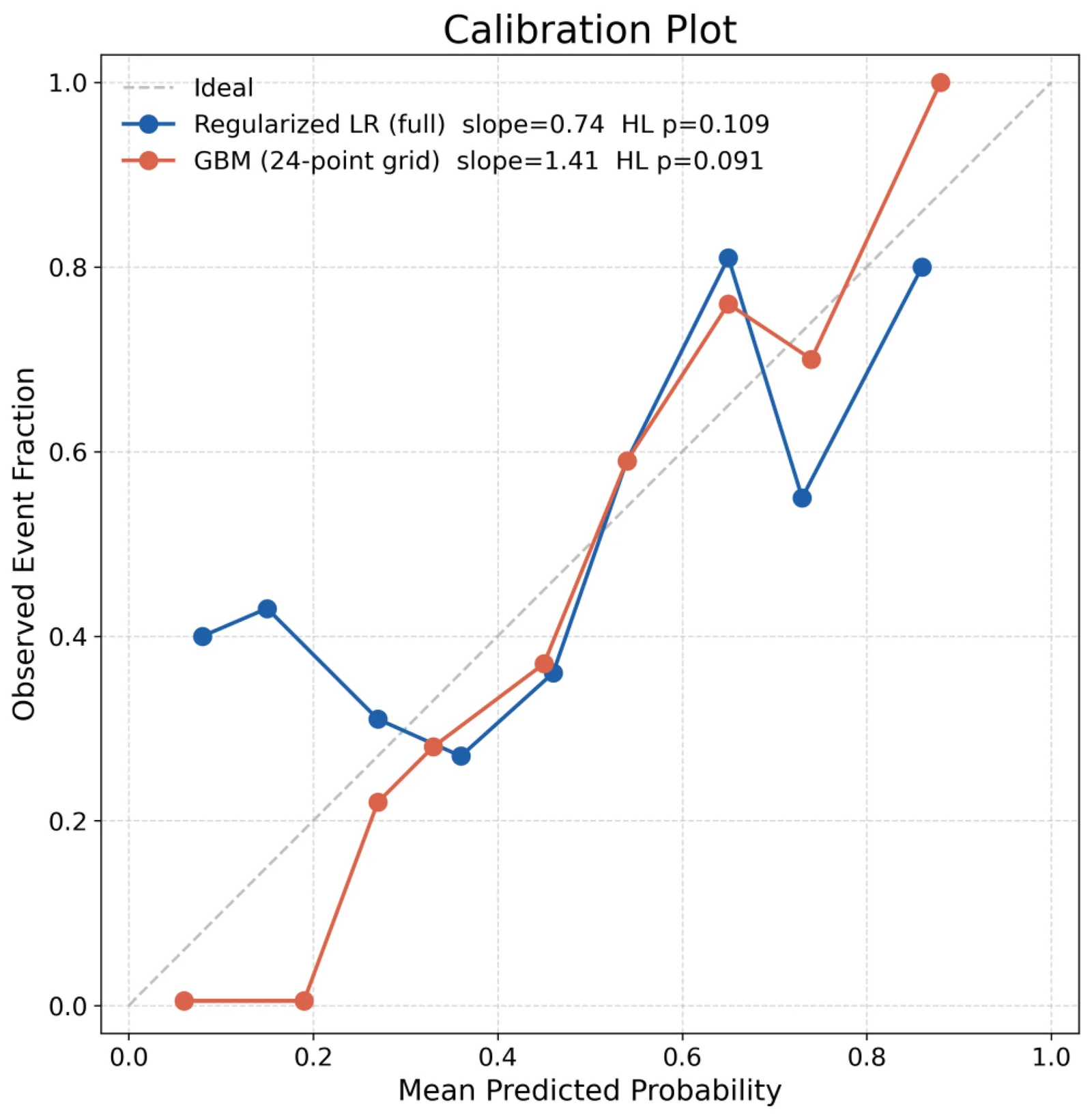
Calibration plots (reliability diagrams) for regularized LR and GBM. The predicted probabilities were binned into ten equal-frequency groups. Regularized LR (full) slope = 0.741; GBM (24-point grid) slope = 1.412. Neither model showed significant miscalibration on the Hosmer–Lemeshow test (*p* = 0.109 and 0.091); at this sample size the test has limited power and a non-significant result is not evidence of good calibration. Dashed diagonal line = perfect calibration.

The 24-point GBM comparator achieved discrimination essentially identical to that of the full elastic-net model (AUROC, 0.699; 95% CI, 0.624–0.769; per-fold range, 0.601–0.846). The 0.003 difference in AUROC between the two models was not statistically significant according to DeLong’s test (*p* = 0.924). At the Youden-optimal threshold (0.506), the GBM achieved the same sensitivity as the elastic-net model with lower specificity. Its calibration slope was 1.412, indicating a departure from the ideal value of 1.0, so its predicted probabilities should be interpreted cautiously. The expanded 72-point hyperparameter grid evaluated in the sensitivity analysis yielded an AUROC of 0.715 vs. 0.696, a difference that was also not statistically significant (DeLong *p* = 0.531). Overall, the more flexible nonlinear model provided no measurable improvement in predictive performance in this cohort. The ROC curves are shown in Figure 2, and the calibration plots are shown in Figure 3.

### 4.4. Threshold-Dependent Performance

**Figure 4:**
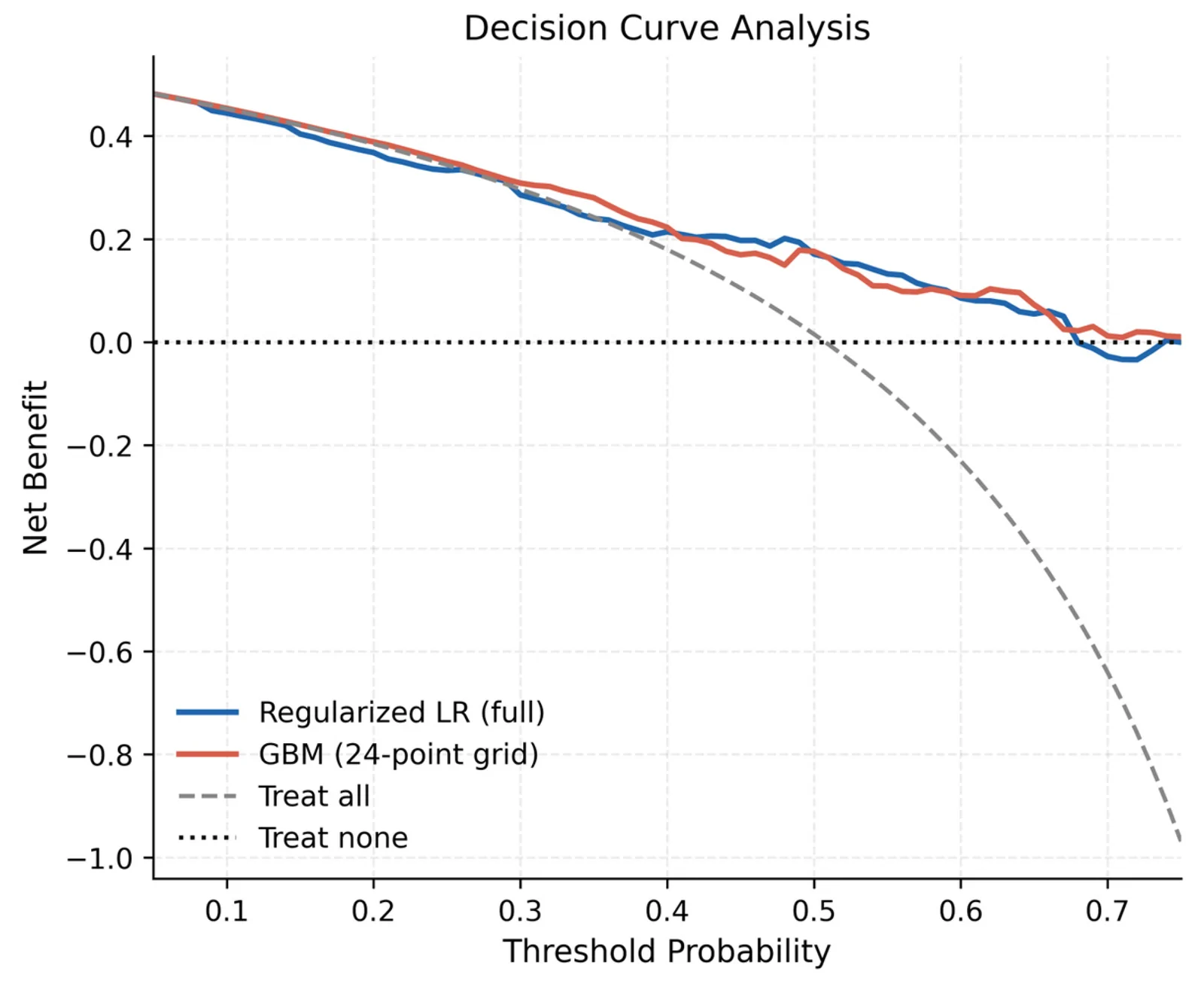
Decision curve analysis across threshold probabilities of 0.05–0.75 using pooled out-of-fold predictions for the full regularized logistic regression model and gradient boosting comparator. The full model exceeded both treat-all and treat-none over a continuous threshold interval of 0.33–0.66 but was not consistently superior at lower threshold values. These internally validated curves are exploratory and do not establish their clinical utility.

At the Youden-optimal threshold, the full elastic-net model achieved a sensitivity of 71.4% and a specificity of 64.2% (Table 4). At its own Youden-optimal threshold (0.506) the gradient boosting model achieved the same sensitivity (71.4%) with slightly lower specificity (62.1%), while its overall AUROC was essentially identical. Accordingly, the GBM identified the same proportion of patients who developed delirium but generated slightly more false-positive classifications among patients who did not. The decision curve analysis across the evaluated threshold probabilities is presented in Figure 4.

### 4.5. Performance of the Reduced Model

The fixed reduced model demonstrated higher apparent internal discrimination than the full model did. The AUROC increased from 0.696 for the full model to 0.756 (95% CI, 0.684–0.819), representing an absolute improvement of approximately 6 percentage points. Fold-to-fold variability was also lower for the reduced model (SD, 0.056 vs. 0.085), and the AUROC difference was statistically significant according to DeLong’s test (*p* = 0.006). The Brier score decreased from 0.228 in the full model to 0.205 in the reduced model. Because the Brier score reflects both discrimination and calibration, this improvement should not be interpreted as evidence of better calibration. The reduced model exhibited a substantially poorer calibration slope.

This pattern is consistent with the higher nominal EPV of the reduced model (8.6 vs. 1.96). However, these findings should be regarded as exploratory rather than confirmatory because the reduced predictor set was derived from the same development dataset used for internal validation, potentially leading to overly optimistic performance estimates. Accordingly, the reduced model should be considered an internally derived candidate model that requires external validation before its performance can be generalized to independent cohorts.

However, calibration remains a concern. The calibration slope was 0.235, and the Hosmer–Lemeshow test was borderline (*p* = 0.056). However, given the present sample size, the Hosmer–Lemeshow test has limited power and should not be interpreted as evidence of good calibration. These findings suggest that the predicted probabilities may not provide reliable estimates of the absolute individual risk. Poor calibration may reflect a combination of small training folds, strong regularization, and isotonic calibration in a limited dataset. For comparison, applying Platt scaling to the same out-of-fold predictions yields a calibration slope of 1.000 by construction and therefore should not be interpreted as evidence of a generalizable calibration. Overall, the reduced model appears to be better suited for ranking patients according to relative risk than for estimating absolute individual risk without external recalibration.

Nested feature selection sensitivity analysis. When the bootstrap feature selection procedure was repeated independently within each outer training fold, the nested reduced model achieved an AUROC of 0.700 (95% CI: 0.623–0.773). This performance was essentially identical to that of the full model (AUROC, 0.696; DeLong *p* = 0.87) but was significantly lower than that of the fixed reduced model (AUROC, 0.756; DeLong *p* = 0.007). Only three predictors—vasopressor use, nasogastric tube, and albumin—were selected from all five outer folds. These findings suggest that the apparent advantage of the fixed reduced model was largely attributable to feature selection on the same development dataset used for evaluation and that predictor stability remained limited under low EPV conditions.

Bootstrap confidence intervals for calibration remained large. The calibration slope was 0.741 (95% CI, 0.345–1.265) for the full model and 0.235 (95% CI, 0.068–0.886) for the fixed reduced model, supporting the conclusion that the reduced model tended to produce overly extreme predicted probabilities, despite substantial uncertainty. Removing SOFA and APACHE II from the reduced model did not reduce discrimination (AUROC, 0.764 vs. 0.756; DeLong *p* = 0.44), indicating that these composite severity scores added no measurable discriminative value beyond individual predictors. The Brier score difference between the reduced and full models was modest (−0.023; 95% CI, −0.039 to −0.004; *p* = 0.02). Confusion matrices are provided in Supplementary Table S6, and the complete sensitivity analyses are presented in Supplementary Table S7.

### 4.6. Feature Importance

**Figure 5:**
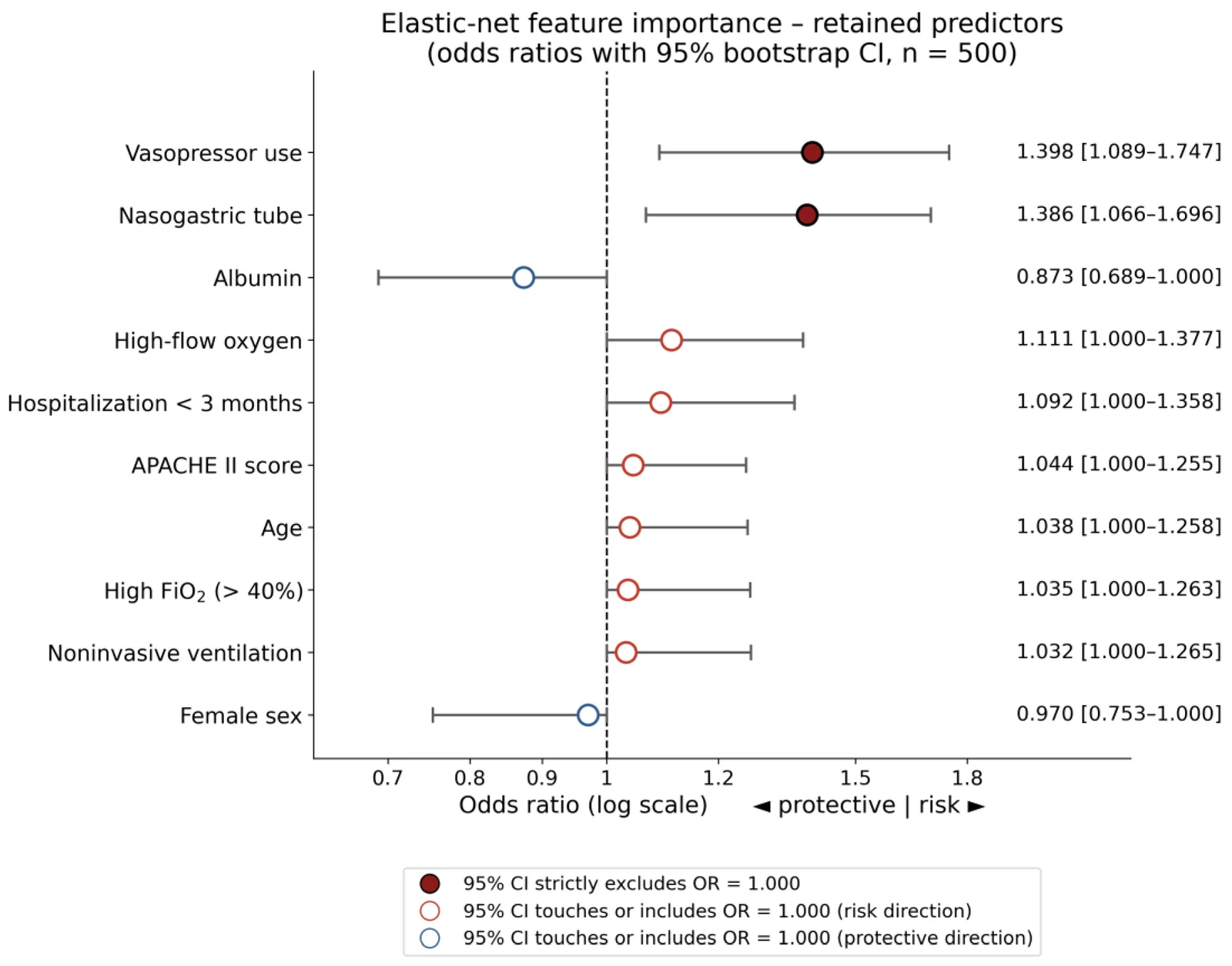
Feature importance for the full elastic-net regularized logistic regression model, shown as odds ratios on a logarithmic axis with the null at OR = 1.000. Points are odds ratios and horizontal bars are 95% bootstrap confidence intervals (500 resamples). The right-hand column repeats each estimate to three decimal places. Filled markers indicate the two predictors whose intervals strictly exclude OR = 1.000 (vasopressor use and nasogastric tube); open markers indicate intervals that touch or include 1.000. The plot shows the 10 non-diagnosis predictors that the penalty retained; the L1 component shrank the remaining 39 non-diagnosis predictors and all admission diagnosis one-hot columns to exactly zero (OR = 1.000), which are listed in Supplementary Table S5. Axis limits contain every confidence interval without truncation.

Table 5 summarizes the predictors included in the fixed reduced model together with their regularized odds ratios and 95% bootstrap confidence intervals estimated from the development dataset. The strongest positive associations were observed for vasopressor use (OR, 1.398; 95% CI, 1.089–1.747) and nasogastric tube placement (OR, 1.386; 95% CI, 1.066–1.696). Higher albumin levels were associated with lower odds of delirium (OR, 0.873; 95% CI: 0.689–1.000). In comparison, recent hospitalization (OR, 1.092; 95% CI, 1.000–1.358) and female sex (OR, 0.970; 95% CI, 0.753–1.000) showed effect estimates closer to the null. The remaining predictors had smaller regularized effect estimates, with bootstrap confidence intervals approaching or crossing an odds ratio of 1.0. These regularized odds ratios should be interpreted as descriptive measures of feature importance within the fitted model, rather than as independently validated effect estimates. The corresponding feature importance forest plot is presented in Figure 5. The complete coefficient table for all encoded predictors, including one-hot encoded admission diagnosis variables, and the model equation are provided in Supplementary Table S5.

### 4.7. Domain Ablation Analysis

**Figure 6:**
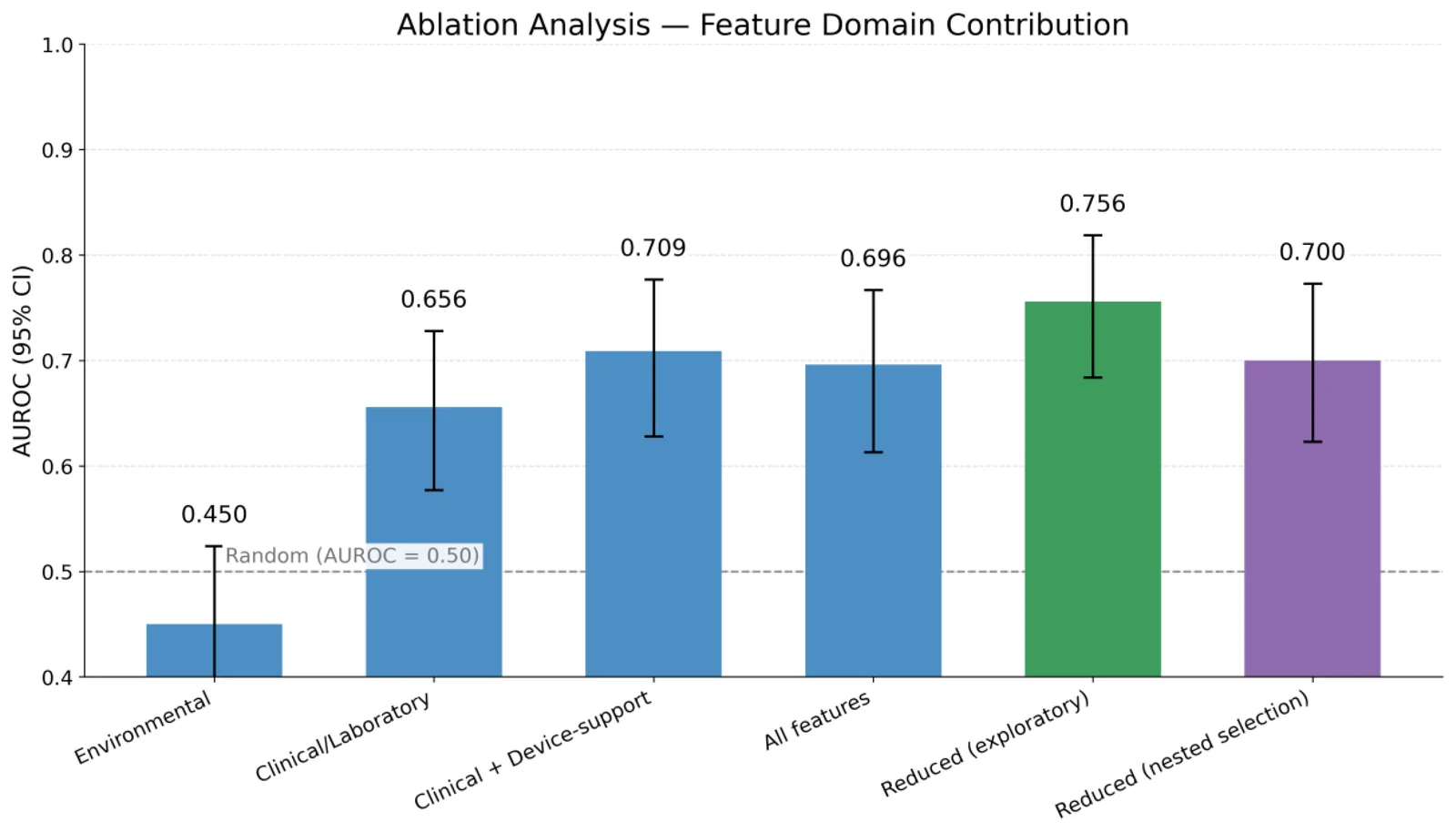
Ablation analysis: AUROC with 95% bootstrap CIs for the four feature-domain models plus the exploratory and nested reduced models. Device-support features provided the largest marginal gain (+5.2 points over clinical/laboratory alone). Environmental features alone did not exceed chance-level performance (AUROC 0.450; permutation *p* = 0.117). The panel also shows the reduced model under nested feature selection (AUROC 0.700) alongside the exploratory reduced model (0.756).

Ablation analysis indicated that the device-support variables provided predictive information beyond the clinical/laboratory feature set. Including device-support variables increased the AUROC by 5.2 percentage points compared with the clinical/laboratory model, from 0.656 (95% CI, 0.577–0.728) to 0.709 (95% CI, 0.628–0.777) (Table 6). In contrast, environmental variables alone showed no useful discriminative ability, with an AUROC of 0.450 (95% confidence interval [CI], 0.369–0.524). Because the AUROC was below 0.50, a one-sided permutation test was performed to determine whether this reflected a reproducible inverse association, rather than random variation. The result was not statistically significant (2000 permutations; *p* = 0.117), indicating that the environmental model was compatible with chance-level performance. The ablation results are presented in Figure 6.

### 4.8. Exploratory Sensitivity Analyses

Exploratory sensitivity analyses were performed to assess the robustness of the main findings to potential outcome misclassifications. Table 7 presents the decision curve results at the selected, clinically relevant thresholds. Although CAM-ICU assessments were performed twice daily, brief or predominantly hypoactive delirium episodes may have been missed. To examine the potential impact of this limitation, we simulated outcome misclassification by randomly changing 5–20% of the negative labels to positive labels. For each level of simulated label noise, the full nested cross-validation pipeline was rerun 50 times and evaluated against the original labels. Discrimination remained relatively stable across simulated scenarios. At 10% simulated noise, the mean AUROC was 0.705 ± 0.027, compared with 0.696 for the original model, whereas at 20% simulated noise, the mean AUROC was 0.686 ± 0.030. Under this single hypothetical mechanism, discrimination varied only modestly across simulated scenarios. This analysis did not estimate or bound the effect of actual outcome misclassification in the study cohort.

The predicted risks were further examined across the device burden strata. The device burden score was calculated as the sum of the eight device-support variables, with the two ordinal items taken at face value. Patients in the high-burden group (score ≥ 4, *n* = 78) had a delirium prevalence of 74.4% and a mean predicted probability of 0.722, whereas those in the low-burden group (score 0–1, *n* = 33) had corresponding values of 21.2% and 0.202. The observed-to-predicted ratios were 1.05, 0.96, and 1.03 in the low-, medium-, and high-burden groups, respectively (Table 8). The predicted probabilities generally tracked the observed increase in delirium prevalence across the device burden strata. However, this should be interpreted as a coarse subgroup calibration assessment, rather than evidence of reliable individual-level calibration.

Table 9 summarizes the reduced model sensitivity analyses. The primary analysis was the nested feature selection procedure described in Section 4.5. When feature selection was repeated independently within each outer training fold, the reduced model achieved an AUROC of 0.700, which was similar to that of the full model (0.696; DeLong’s *p* = 0.87). In contrast, the fixed reduced model, which reused the full development dataset for feature selection, retained an AUROC of 0.756 (DeLong *p* = 0.007 vs. the nested estimate). Only vasopressor use, nasogastric tube placement, and albumin were selected from all five outer folds.

## 5. Discussion

### 5.1. Principal Findings

In this single-center model development study, regularized logistic regression showed moderate performance in predicting ICU delirium. While the full model achieved an AUROC of 0.696, the fixed reduced model showed higher discrimination (AUROC, 0.756). This apparent difference is consistent with the low nominal EPV of the complete model. Penalized regression can mitigate coefficient instability when the number of candidate predictors is high relative to the number of outcome events; however, it cannot compensate for the limited outcome information. However, this apparent advantage did not persist in the nested feature selection sensitivity analysis (AUROC, 0.700 vs. 0.696; DeLong *p* = 0.87), indicating that the fixed reduced model should be regarded as exploratory rather than confirmatory (Section 4.5; Table 9).

The reduced model demonstrated more stable performance during internal validation. However, these findings should be interpreted with caution. Because the reduced predictor set was selected from the same development dataset used for internal validation, the apparent performance advantage may reflect selection-induced bias. Although the AUROC difference was statistically significant in the DeLong test (*p* = 0.006), this does not imply that the reduced model would retain the same advantage in an independent cohort. Accordingly, the reduced model should be regarded as an exploratory candidate that requires external validation. Consistent with this interpretation, the nested feature selection sensitivity analysis showed no meaningful difference between the reduced and full models (AUROC: 0.700 vs. 0.696; DeLong *p* = 0.87).

Calibration was the principal weakness across the developed models, although it differed between them. The fixed reduced model produced overly extreme predicted probabilities (calibration slope, 0.235), a pattern expected when a model is overfitted to its development data. For the full model the calibration slope was 0.741 with a wide confidence interval that included 1.0 (0.345–1.265), so its calibration is imprecise rather than demonstrably poor. The GBM comparator showed a slope of 1.412, a departure in the opposite direction. Therefore, its predicted probabilities should not be interpreted as reliable estimates of the individual absolute risk without recalibration. Poor calibration may reflect the combined effects of small training folds, strong regularization, and isotonic calibration in a limited dataset. Post hoc recalibration of the same out-of-fold predictions yielded a calibration slope of 1.000 by construction and therefore should not be interpreted as evidence of generalizable calibration. At best, the fixed reduced model may support exploratory relative-risk ranking rather than absolute-risk estimation, as its apparent AUROC advantage disappears under nested feature selection. None of the developed models are ready for clinical use.

### 5.2. Clinical Interpretation of Predictor Domains

In the ablation analysis, adding device-support variables to the clinical and laboratory predictors improved the model performance. The AUROC increased by approximately 5.2 percentage points when the device-support domain was included. These findings do not imply that device support variables should be used in isolation; rather, they suggest that routinely collected support-related information provides incremental value for delirium risk stratification. In clinical practice, identifying a patient as high-risk would primarily support intensification of routine non-pharmacological prevention (e.g., prioritized early mobilization, lighter and regularly reviewed sedation, sleep preservation, and family engagement) rather than pharmacological treatment. The threshold range (0.05–0.75) examined in the decision curve analysis was selected to represent plausible clinician willingness to provide these additional, low-risk but resource-intensive preventive interventions.

The most prominent device-related predictors were vasopressor use and nasogastric tube placement. These variables should not be interpreted as evidence that the devices directly cause delirium but rather as markers of disease severity and intensity of care. The need for vasopressors reflects hemodynamic deterioration and more severe acute illnesses. Nasogastric tube placement may indicate impaired oral intake, restricted mobility, neurological vulnerability, and conditions that require intensive nursing care. High-flow oxygen use and FiO_2_ > 40% likely reflect more severe respiratory failure, consistent with the established association between hypoxemia and delirium [6]. Because removing SOFA and APACHE II from the reduced model did not reduce discrimination (AUROC, 0.764 vs. 0.756; DeLong *p* = 0.44), a simpler set of directly observable variables may offer a more practical bedside model for predicting delirium. However, this hypothesis requires external validation.

Higher albumin levels were associated with lower delirium odds. Low serum albumin levels have been associated with delirium in observational ICU cohorts and may reflect the combined effects of systemic inflammation, malnutrition, and acute illness burden [27,28]. Although this association is consistent with previous observational studies, it was identified in a single cohort. The predictive value of albumin should be confirmed in independent external cohorts before it can be regarded as an independently confirmed predictor of ICU delirium.

Environmental variables did not provide meaningful discrimination when used independently of other predictors. The environmental domain included noise exposure, family visits, television/radio/tablet use, newspaper or magazine access, and visual stimulation. The environmental-only model achieved an AUROC below 0.50; however, the permutation test indicated that this result was compatible with chance-level performance rather than a reproducible, inverse association. This should not be interpreted as evidence that environmental interventions are ineffective. The available environmental variables were recorded only as binary presence/absence indicators and therefore may not have captured the intensity, duration, timing, or exposure quality. Their effects may also depend on the sedation level, sleep disruption, illness severity, and baseline vulnerability. A more plausible interpretation is that these routinely documented environmental variables lacked sufficient granularity to serve as useful, stand-alone predictors in this cohort.

### 5.3. Methodological Considerations

Across the outer folds, the elastic-net model most frequently selected a balanced penalty (l1-ratio = 0.5, C = 0.05). This combination encourages sparse models while stabilizing the coefficient estimates. For this dataset, such a balance was appropriate, given the low nominal EPV and relatively large number of candidate predictors. However, although penalization can improve model stability under these conditions, it cannot compensate for limited outcome information, as demonstrated by the nested feature selection sensitivity analysis.

However, the coefficient patterns should not be overinterpreted. In a low-EPV setting, small changes in the development sample may alter which predictors are retained after penalization and which are reduced to zero. Therefore, the elastic-net model is best viewed as describing the multivariable prediction pattern within this development cohort rather than defining a definitive set of independently validated predictors of delirium.

The same caution applies to the fixed-reduced model. Although it showed higher apparent discrimination and lower fold-to-fold variability than the full model, the predictor set was derived from the same development dataset used for evaluation, making its apparent performance susceptible to selection-induced bias. The interpretation is further limited by the poor calibration of the model. Post hoc recalibration of the same out-of-fold predictions may be useful as a diagnostic exercise but cannot substitute for recalibration and external validation in independent cohorts.

Gradient boosting analyses did not provide clear evidence that a more flexible nonlinear model offered superior predictive performance to regularized logistic regression in this cohort. The prespecified 24-point GBM comparator showed discrimination essentially identical to that of the full elastic-net logistic regression model, and the AUROC difference was not statistically significant. The expanded 72-point GBM grid produced a numerically higher AUROC than the full logistic regression model, but this difference was also not statistically significant. Its calibration slope departed further from 1.0 than that of the elastic-net model, so the added flexibility did not translate into better-calibrated probabilities either. Accordingly, the expanded GBM analysis should be interpreted as a sensitivity analysis, rather than evidence that gradient boosting outperformed regularized logistic regression.

The comparator models exhibited different sensitivity–specificity trade-offs across the decision thresholds. For example, at its own Youden-optimal threshold the GBM model reached the same sensitivity as the full model with lower specificity. Such operating-point differences could become clinically relevant if a model is externally validated, recalibrated, and integrated into a predefined, preventive care pathway. However, the threshold-based findings of the present study are exploratory and should not be used to define clinical decision rules.

Table 10 compares the present model with representative ICU delirium prediction models. Two patterns are particularly relevant to this study. First, external validation remains uncommon, and when performed, the model discrimination is typically lower than that observed during development. Second, the internally validated performance estimates reported here (AUROC 0.696 for the full model, 0.756 for the fixed reduced model, and 0.700 under nested feature selection) are broadly comparable to the externally validated performance of established models, including the Turkish PRE-DELIRIC validation (AUROC 0.649). Overall, these findings are consistent with the expected performance of a locally developed model that has not yet undergone external validation, rather than indicating its superiority over existing prediction models.

### 5.4. Limitations and Future Validation

The main limitation of this study was the small sample size relative to the number of potential predictors. The full model was developed using 98 events and 50 candidate predictors, yielding a nominal event per variable (EPV) of 1.96. The expansion of the admission diagnosis variable through one-hot encoding further increased the effective model complexity. Under these low information-to-parameter conditions, coefficient estimates are likely to be unstable, and the internal performance estimates may be optimistic. Although the fixed reduced model increased the nominal EPV and showed improved apparent performance, these findings remain susceptible to selection-induced optimism because the predictor selection and evaluation were performed within the same development dataset. Therefore, external validation is required before any apparent performance advantage can be considered generalizable.

The generalizability of the present findings is limited. This study was conducted in a single tertiary ICU in Türkiye. Local factors, including case mix, staffing structure, documentation practices, sedation protocols, and delirium assessment routines, may have influenced both the model performance and the predictive value of individual predictors. Without external validation, the performance of the model in other ICU settings remains uncertain.

Calibration remains the principal limitation of the proposed models. The fixed reduced model achieved higher apparent internal discrimination, but its predicted probabilities were too extreme and are unsuitable for individual absolute-risk estimation; for the full model the calibration slope estimate was imprecise, with a confidence interval that included 1.0. External validation, with recalibration as required in the target population, is necessary before clinical implementation.

Outcome misclassification cannot be excluded from this study. Delirium was assessed twice daily during morning and evening physician rounds using a structured delirium screening checklist and CAM-ICU. Although this approach reflects routine ICU practice, brief, fluctuating, or predominantly hypoactive delirium episodes may have been missed during scheduled assessments. Formal inter-rater agreement was not quantified because independent paired assessments were not conducted. Although suspected cases were reviewed by the attending intensivist, this clinical workflow does not substitute for a formal inter-rater reliability study. Accordingly, the label-noise sensitivity analysis should be interpreted only as an exploratory assessment of one hypothetical mechanism in which negative labels were converted to positive labels. It neither estimates nor bounds the magnitude of actual outcome misclassification.

Residual temporal leakage could not be completely excluded. Predictors were restricted to variables available at ICU admission or within the first 24 h of ICU stay, and variables occurring after delirium onset were excluded. However, in patients with very early delirium, the timing of some device support variables relative to symptom onset could not be fully reconstructed, meaning that certain exposures may have been recorded close to or after the first signs of delirium. Because the dataset did not record either the timing of the first CAM-ICU-positive assessment or the initiation times of device-supported interventions, a delirium-free prediction landmark could not be defined. Consequently, the model should be interpreted as providing risk stratification based on variables documented during the first 24 h of ICU admission, rather than time-anchored risk prediction. Future studies should incorporate a prespecified prediction landmark and prospectively record the timing of both predictor measurements and the onset of delirium.

Several clinically important predictors were unavailable in this dataset, including detailed sedation depth, benzodiazepine and opioid exposure, anticholinergic burden, sleep disruption, pain scores, and longitudinal RASS trajectories. These variables are well-established determinants of ICU delirium, and their absence may partly explain the apparent predictive contribution of device-support variables, which may act as proxies for unmeasured sedation, analgesia, organ-support intensity, and overall care complexity. In addition, comorbidity was recorded only as none, one, or two or more conditions rather than using a validated index such as the Charlson Comorbidity Index, limiting comparability with previous studies. Future studies should prospectively collect longitudinal sedation depth (RASS) and medication exposure data to strengthen both model development and external validation.

Temporal validation could not be performed because admission timestamps were available for only 46 of the 193 patients included in the study. Future datasets should record admission timestamps separately from clinical predictors to enable temporal validation while avoiding information leakage. Larger prospective, preferably multicenter cohorts are needed to externally validate the reduced model, recalibrate the predicted probabilities where necessary, and determine whether the predictive value of the device support burden is consistent across different ICU populations and care settings.

## 6. Conclusions

In this single-center Turkish ICU cohort, regularized logistic regression achieved moderate discrimination during internal validation for ICU delirium prediction. The fixed reduced model showed higher apparent discrimination than the full model; however, this advantage disappeared when feature selection was repeated within each training fold, indicating that the apparent improvement was largely attributable to selection-induced optimism. Device-support burden provides predictive information beyond routinely available clinical and laboratory variables and is best interpreted as a marker of organ-support intensity and overall care complexity rather than a direct causal factor. In contrast, the available binary environmental indicators did not provide meaningful stand-alone discrimination results. The inadequate calibration of the fixed reduced model, together with the small single-center sample, low EPV, and lack of external validation, indicate that the model is not yet ready for clinical implementation. Larger prospective multicenter studies are required for external validation and recalibration and should include more detailed information on sedation, analgesia, and comorbidities. Because the timing of delirium onset and device support initiation was unavailable, the model should be interpreted as stratifying delirium risk using variables documented at ICU admission or within the first 24 h rather than as a landmark-based prediction model.

## Figures and Tables

**Table 1:** Complete Predictor Variable List (*n* = 50).

#	Variable	Domain	Type	Coding
1	Age	Demographic	Continuous	Years
2	Sex	Demographic	Binary	0 = male, 1 = female
3	Admission diagnosis	Clinical	Categorical	One-hot encoded
4	SOFA score	Severity	Ordinal	0–24
5	APACHE-II score	Severity	Ordinal	0–71
6	Fever (>37.5 °C)	Clinical	Binary	0 = no, 1 = yes
7	Prior hospitalization (3 mo)	Clinical	Binary	0 = no, 1 = yes
8	Comorbidity burden	Clinical	Ordinal	0 = none, 1 = one, 2 = ≥two
9	Malignancy history	Clinical	Binary	0 = no, 1 = yes
10	ABO blood group	Clinical	Ordinal	0 = O, 1 = A, 2 = B, 3 = AB
11	Rh factor	Clinical	Binary	0 = Rh−, 1 = Rh+
12	Positive blood culture	Clinical	Binary	0 = no, 1 = yes
13	Polypharmacy (≥5 regular drugs)	Clinical	Binary	0 = no, 1 = yes
14	Blood glucose	Laboratory	Continuous	mg/dL
15	pH	Laboratory	Continuous	Units
16	pCO_2_	Laboratory	Continuous	mmHg
17	HCO_3_	Laboratory	Continuous	mEq/L
18	Lactate	Laboratory	Continuous	mmol/L
19	WBC	Laboratory	Continuous	×10^3^/μL
20	Hemoglobin	Laboratory	Continuous	g/dL
21	Hematocrit	Laboratory	Continuous	%
22	Platelets	Laboratory	Continuous	×10^3^/μL
23	Neutrophils	Laboratory	Continuous	×10^3^/μL
24	Lymphocytes	Laboratory	Continuous	×10^3^/μL
25	Eosinophils	Laboratory	Continuous	×10^3^/μL
26	BUN	Laboratory	Continuous	mg/dL
27	Creatinine	Laboratory	Continuous	mg/dL
28	Sodium	Laboratory	Continuous	mEq/L
29	Chloride	Laboratory	Continuous	mEq/L
30	Potassium	Laboratory	Continuous	mEq/L
31	Magnesium	Laboratory	Continuous	mg/dL
32	CRP	Laboratory	Continuous	mg/L
33	Procalcitonin	Laboratory	Continuous	ng/mL
34	Albumin	Laboratory	Continuous	g/L
35	Uric acid	Laboratory	Continuous	mg/dL
36	High FiO_2_ (>40%)	Clinical	Binary	0 = no, 1 = yes
37	Mechanical ventilation (MV)	Device-support	Ordinal	0 = no, 1 = yes, 2 = tracheostomy
38	Noninvasive ventilation (NIMV)	Device-support	Binary	0 = no, 1 = yes
39	High-flow oxygen (HFO)	Device-support	Binary	0 = no, 1 = yes
40	Vasopressor	Device-support	Binary	0 = no, 1 = yes
41	Renal replacement therapy (RRT)	Device-support	Binary	0 = no, 1 = yes
42	Central venous catheter (CVC)	Device-support	Binary	0 = no, 1 = yes
43	Nasogastric tube (NG tube)	Device-support	Ordinal	0 = no, 1 = NG, 2 = PEG
44	Urinary catheter	Device-support	Binary	0 = no, 1 = yes
45	Family visit (≥1 h/day)	Environmental	Binary	0 = no, 1 = yes
46	Intolerable noise exposure	Environmental	Binary	0 = no, 1 = yes
47	Radio/tablet use	Environmental	Binary	0 = no, 1 = yes
48	Television use	Environmental	Binary	0 = no, 1 = yes
49	Newspaper/magazine access	Environmental	Binary	0 = no, 1 = yes
50	Excessive visual stimulation	Environmental	Binary	0 = no, 1 = yes

Features were extracted at or within 24 h of the ICU admission. Compound EMR fields (blood gas, CBC differential, electrolytes, albumin/uric acid, CRP/PCT) were parsed from slash-delimited strings. SOFA, Sequential Organ Failure Assessment; APACHE, Acute Physiology and Chronic Health Evaluation; BUN, blood urea nitrogen; CRP, C-reactive protein; FiO_2_, fraction of inspired oxygen; MV, mechanical ventilation; NIMV, noninvasive mechanical ventilation; HFO, high-flow oxygen; RRT, renal replacement therapy; CVC, central venous catheter; PEG, percutaneous endoscopic gastrostomy.

**Table 2:** Cohort Characteristics by Delirium Status (*n* = 193).

Variable	Overall (*n* = 193)	Delirium (*n* = 98)	No Delirium (*n* = 95)	Test	*p* (Raw)	*q* (BH-FDR)
Age, years	69 [56–79]	70 [60–80]	66 [49–77]	Mann–Whitney	0.012	0.052 ^†^
Sex, female	89 (46.1%)	37 (37.8%)	52 (54.7%)	Fisher exact	0.021	0.080 ^†^
SOFA score	5 [4–7]	6 [4–7]	4 [3–6]	Mann–Whitney	0.003	0.018 *
APACHE-II score	20 [16–25]	22 [17–25]	18 [15–25]	Mann–Whitney	0.007	0.034 *
Vasopressor	60 (31.1%)	47 (48.0%)	13 (13.7%)	Fisher exact	<0.001	<0.001 *
NG tube	0 [0–1]	1 [0–1]	0 [0–0]	Mann–Whitney	<0.001	<0.001 *
HFO	63 (32.6%)	42 (42.9%)	21 (22.1%)	Fisher exact	0.002	0.016 *
Mechanical ventilation	0 [0–0]	0 [0–1]	0 [0–0]	Mann–Whitney	0.007	0.034 *
NIMV	86 (44.6%)	54 (55.1%)	32 (33.7%)	Fisher exact	0.004	0.021 *
High FiO_2_ (>40%)	79 (40.9%)	51 (52%)	28 (29.5%)	Fisher exact	0.001	0.013 *
Prior hospitalization	109 (56.5%)	67 (68.4%)	42 (44.2%)	Fisher exact	0.001	0.013 *
CRP, mg/L	73 [33–176]	95 [51–189]	54 [20–139]	Mann–Whitney	0.002	0.013 *
Albumin, g/L	30.0 [25.5–34.2]	28.5 [25–32.5]	32.0 [27–36.0]	Mann–Whitney	0.001	0.013 *
Comorbidity count	2 [1–2]	2 [1–2]	2 [1–2]	Mann–Whitney	0.038	0.128 ^†^
Blood urea nitrogen	78 [47–123]	89 [55–133]	70 [39–111]	Mann–Whitney	0.022	0.080 ^†^

Values are median [IQR] for continuous/ordinal variables (including the three-level ordinal device variables MV and NG tube); binary variables are reported as *n* (%). Tests: Fisher exact for binary variables; Mann–Whitney U for continuous/ordinal; χ^2^ for multicategory. * Significant at BH-FDR *q* < 0.05. ^†^ Significant at raw *p* < 0.05 only (does not survive FDR correction). Only variables with *p* < 0.05 are shown. SOFA, Sequential Organ Failure Assessment; APACHE, Acute Physiology and Chronic Health Evaluation; HFO, high-flow oxygen; NIMV, noninvasive mechanical ventilation; NG, nasogastric; CRP, C-reactive protein; IQR, interquartile range. A complete comparison of all 50 candidate predictors, with raw *p*-values and Benjamini–Hochberg *q*-values, is provided in Supplementary Table S3.

**Table 3:** Model Performance Summary (Bootstrap *n* = 1000).

Model	AUROC (95% CI)	AUPRC (95% CI)	Brier Score	Cal. Slope	HL *p*
Regularized LR—full (50 features)	0.696 (0.613–0.767)	0.689 (0.591–0.782)	0.228 (0.206–0.254)	0.741	0.109
Regularized LR—reduced (11 features)	0.756 (0.684–0.819)	0.743 (0.650–0.839)	0.205 (0.177–0.238)	0.235 ^‡^	0.056
GBM comparator—24-point grid (50 features)	0.699 (0.624–0.769)	0.700 (0.608–0.790)	0.220 (0.204–0.236)	1.412	0.091
GBM comparator—expanded 72-point grid	0.715 (0.642–0.786)	0.710 (0.612–0.806)	0.214 (0.196–0.233)	1.312	0.411

Bootstrap 95% CIs were calculated using the percentile method. Cal. slope: ideal = 1.0. HL = Hosmer–Lemeshow, df = 8; a non-significant *p*-value reflects limited power at this sample size and is not evidence of good calibration. ^‡^ A calibration slope of 0.235 indicates that the reduced model’s predicted probabilities are far too extreme; they must be recalibrated before any clinical use. Fitting Platt scaling to the same out-of-fold predictions returns a slope of 1.000 by construction and is not independent evidence of calibration (Section 5.1). DeLong: reduced vs. full, *p* = 0.006; full vs. 24-point GBM, *p* = 0.924; full vs. expanded GBM, *p* = 0.531. Bootstrap 95% CIs for the calibration slope: full model, 0.741 (0.345–1.265); reduced model, 0.235 (0.068–0.886).

**Table 4:** Threshold-Dependent Performance at the Youden-Optimal Threshold.

Model	Threshold	TP	FN	TN	FP	Sensitivity	Specificity	PPV	NPV	F1	Accuracy
Regularized LR	0.497	70	28	61	34	71.4%	64.2%	67.3%	68.5%	0.693	67.9%
GBM comparator	0.506	70	28	59	36	71.4%	62.1%	66.0%	67.8%	0.686	66.8%

Youden index = sensitivity + specificity − 1. The threshold performance of the reduced model was not separately listed. The AUROC of 0.756 indicates a more advantageous operating curve at most thresholds, but this apparent advantage does not persist under nested feature selection (AUROC 0.700; Table 9), so it should be read as exploratory. Confusion matrix counts (TP, true positives; FN, false negatives; TN, true negatives; FP, false positives) were computed from the pooled out-of-fold predictions (*n* = 98 with delirium, 95 without delirium) and are provided in Supplementary Table S6.

**Table 5:** Predictors Retained in the Exploratory Reduced Model.

Feature	Domain	OR	95% CI	Direction
Vasopressor	Device-support	1.398	1.089–1.747	↑ Risk
NG tube	Device-support	1.386	1.066–1.696	↑ Risk
Albumin	Laboratory	0.873	0.689–1.000	↓ Risk
Prior hospitalization (3 mo)	Clinical	1.092	1.000–1.358	↑ Risk
Sex (female)	Demographic	0.970	0.753–1.000	↓ Risk
HFO	Device-support	1.111	1.000–1.377	↑ Risk
High FiO_2_	Clinical	1.035	1.000–1.263	↑ Risk
APACHE-II	Severity	1.044	1.000–1.255	↑ Risk
Age	Demographic	1.038	1.000–1.258	↑ Risk
SOFA	Severity	1.000	1.000–1.180	↑ Risk
NIMV	Device-support	1.032	1.000–1.265	↑ Risk

ORs from Elastic-Net logistic regression coefficients (modal penalty: l1_ratio = 0.5, C = 0.05) on standardized features estimated on a pre-encoded fixed feature matrix to avoid OHE column-set drift. 95% CIs from 500-resample bootstrap (seed = 42). In the L1 component of the Elastic-Net penalty, most coefficients were reduced to zero. Significantly, only vasopressor and nasogastric (NG) tube exhibited confidence intervals that definitively excluded an odds ratio of 1.0 in the re-estimated Elastic Net model. The remaining nine predictors were identified in an earlier bootstrap analysis conducted under pure-ridge regularization (l1_ratio = 0), under which all 11 CIs excluded OR = 1.0; they were retained in the reduced model because that earlier analysis flagged them and they were clinically plausible, but being derived from the development data, these estimates were exploratory (see Section 3.9). Four of the 11 retained predictors (vasopressor, nasogastric tube, high-flow oxygen, and noninvasive ventilation) belonged to the device-support domain; high FiO_2_ was classified as a clinical/respiratory-support variable, consistent with Table 1 and the ablation domain definition. OR: odds ratio; HFO: high-flow oxygen; NIMV: noninvasive mechanical ventilation; FiO_2_: fraction of inspired oxygen; ↑, increased odds of delirium (OR > 1); ↓, decreased/protective odds of delirium (OR < 1).

**Table 6:** Ablation Analysis: Discriminative Performance by Feature Domain.

Feature Subset	*n* Features	AUROC (95% CI)	AUPRC	Brier
Environmental only	6	0.450 (0.369–0.524)	0.478	0.263
Clinical/Laboratory only	36	0.656 (0.577–0.728)	0.616	0.233
Clinical + Device-Support	44	0.709 (0.628–0.777)	0.698	0.220
All features (full model)	50	0.696 (0.613–0.767)	0.689	0.228
**Reduced** (**exploratory**)	**11**	**0.756** (**0.684–0.819**)	**0.743**	**0.205**

All models: identical 5-fold outer × 4-fold inner nested CV. Bootstrap CIs: 500 resamples. The reduced model is listed separately as a secondary analysis, not as an ablation subset, and is shown in bold to visually distinguish it from the four feature-domain ablation subsets above. The Clinical/Laboratory subset comprised demographic, clinical, illness-severity, and laboratory variables.

**Table 7:** Decision Curve Analysis at Clinically Relevant Thresholds (reduced model).

Threshold	NB Model	NB Treat-All	Treated/100 (Model)	Treated/100 (Treat-All)	Net Reduction in Interventions per 100
0.20	0.396	0.385	82.4	100.0	4.7
0.25	0.363	0.344	79.8	100.0	5.7
0.30	0.337	0.297	75.1	100.0	9.3
0.35	0.301	0.243	71.5	100.0	10.7
0.40	0.261	0.180	68.4	100.0	12.2
0.50	0.192	0.016	59.6	100.0	17.6

NB = net benefit (treat-none NB = 0 at all thresholds). Net reduction in interventions per 100 patients = (NB_model − NB_treat-all) ÷ (pt/(1 − pt)) × 100, the standard decision-curve expression for how many interventions per 100 patients the model avoids at the same net benefit as treating all patients. The earlier version of this column reported (treated per 100 under treat-all) − (treated per 100 by the model), which is the reduction in all interventions rather than in unnecessary ones, and has been corrected in the current version. These values were derived from the exploratory fixed reduced model and should not be interpreted as evidence of clinical benefit.

**Table 8:** Device Burden Subgroup Analysis (reduced model).

Device Burden Group	*N*	Delirium Prevalence (%)	Mean Predicted Probability
Low (score 0–1)	33	21.2	0.202
Medium (score 2–3)	82	40.2	0.418
High (score 4+)	78	74.4	0.722

Device burden score = sum of eight device-support variables (MV, NIMV, HFO, RRT, CVC, NG tube, urinary catheter, and vasopressor; ordinal items at face value). An observed/predicted ratio near 1.0 indicates calibration-in-the-large, within each stratum.

**Table 9:** Reduced-Model Sensitivity Analyses under Nested Feature Selection.

Analysis	Result	Interpretation
Reduced model, selection on the full dataset (exploratory)	AUROC 0.756 (95% CI: 0.684–0.819)	Optimistic: predictors were selected on the evaluation data
Reduced model, feature selection nested within each fold	AUROC 0.700 (95% CI: 0.623–0.773)	Equivalent to the full model (DeLong *p* = 0.87); significantly below the fixed reduced model (DeLong *p* = 0.007)
Predictors selected in all five folds	Vasopressor, nasogastric tube, albumin	Only 3 of 11 stable (6–9 predictors selected per fold), indicating selection instability under low EPV
Untuned baseline (no grid search)	AUROC 0.627 (95% CI: 0.544–0.710)	Reference point only; the baseline also differs in penalty type (L2 at C = 1.0) and lacks isotonic calibration, so the gap cannot be attributed to hyperparameter tuning alone
Reduced model without SOFA and APACHE II	AUROC 0.764 (95% CI: 0.695–0.827)	No loss relative to 0.756 (DeLong *p* = 0.44); the composite scores add no measurable discrimination
Brier score, reduced − full	−0.023 (95% CI: −0.039 to −0.004); *p* = 0.02	Lower apparent score; exploratory and affected by fixed feature selection

All analyses used the identical 5-fold outer × 4-fold inner nested cross-validation protocol and pooled out-of-fold predictions. AUROC comparisons used the DeLong test, and the Brier-score difference used a 1000-resample bootstrap. Bootstrap confidence intervals for the calibration slopes of the same models are provided in Supplementary Table S7. EPV, events per variable; CI, confidence interval.

**Table 10:** Comparison with Representative ICU Delirium Prediction Models.

Model (Ref.)	Cohort/Setting	*n*	Validation	Model Type	AUROC (Development)	AUROC (External)
PRE-DELIRIC [16]	Dutch multicenter ICU	1613	Development + external	Logistic regression	0.87	0.71–0.77 [17,18]
E-PRE-DELIRIC [20]	Multinational ICU	2000	Multinational	Logistic regression	0.76	—
PRIDE [21]	Korean tertiary ICU	large EHR	Internal + external	Machine learning	0.916	0.721
Zhang et al. [29]	MIMIC-IV/eICU-CRD	large	Internal + external	XGBoost	0.793	0.701
Wang et al. [30]	Taiwanese ICU	large	Development + deployment	Machine learning	high internal	—
Turkish PRE-DELIRIC validation [23]	Turkish ICU	172	External validation	Logistic regression (applied)	—	0.649
This study	Turkish internal-medicine ICU	193	Internal (nested 5 × 4 CV, leakage-aware)	Elastic-net logistic regression	0.696 full; 0.756 reduced; 0.700 nested	not yet performed

For the present study, 0.696 is the full 50-predictor model, 0.756 the exploratory reduced model with feature selection on the full dataset, and 0.700 the same reduced model with feature selection nested within each cross-validation fold (Table 9). The predictor counts and further details of the published models are provided in Supplementary Table S4. The development-to-external decreases reported for PRIDE (0.916 → 0.721) and Zhang et al. (0.793 → 0.701) illustrate why the present internal estimates (0.696–0.756) cannot be treated as external performance estimates. EHR, electronic health record; CV, cross-validation; AUROC, area under the receiver operating characteristic curve.

## Data Availability

The de-identified dataset and analysis code can be accessed upon reasonable request from the corresponding author, contingent upon an institutional data-sharing agreement. Because the data are individual-level ICU patient records, public deposition is precluded by ethics approval and applicable data-protection requirements; access is therefore restricted to a reasonable request under an institutional data-sharing agreement.

## Supplementary Materials

The following supporting information can be downloaded at https://www.mdpi.com/article/10.3390/diagnostics16162554/s1: Table S1: Missingness of the 50 candidate predictors considered for model development. Table S2: Admission-diagnosis categories, frequencies and delirium rate. Table S3: Complete baseline comparison for all 50 candidate predictors. Table S4: Extended comparison with previously published ICU delirium prediction models. Table S5: Full coefficient table and mathematical form of the fitted elastic-net logistic regression model. Table S6: Classification metrics and confusion matrix at each model’s Youden-optimal threshold (24-point GBM comparator). Table S7: Model variants and sensitivity analyses.
