# Supplementary material for: Predicting ICU Delirium with a Regularized Logistic Regression Model: Device-Support Burden as an Informative Predictor Domain in a Turkish Cohort
Source: Diagnostics (Basel). 2026 Aug 13;16(16):2554. doi: 10.3390/diagnostics16162554 (PMC13511172; doi:10.3390/diagnostics16162554)
Supplement: Supplementary file 1 [file diagnostics-16-02554-s001.zip › diagnostics-4419610-supplementary.pdf]

Supplementary Material

Predicting ICU Delirium with a Regularized Logistic Regression Model: Device-Support Burden as an Informative Predictor Domain in a Turkish Cohort

Muhammed Sezai Bazna and Fatih Okumuş

Tables S1-S7 accompany the manuscript and are referenced from it. Each table is self-contained: the values shown here are the values reported in the manuscript, and no external file is needed to read them.

Table S1. Missingness of the 50 candidate predictors considered for model development (n = 193)

Feature-level missingness was low: no modelled predictor exceeded 2.07% missing and most were below 1%. Missing values were imputed within each outer training fold only (median for continuous and ordinal variables, most-frequent category for the admission-diagnosis variable), so no imputation parameter was learned from a held-out fold. Outcome, post-outcome, administrative and temporally dependent fields were not part of the candidate predictor set and are therefore not listed here: the delirium outcome itself, delirium subtype, ICU length-of-stay category, post-diagnosis pharmacological treatment, hospital outcome and admission date. Admission date was 76.17% missing and was excluded a priori as a temporal-leakage source rather than imputed, which is also why temporal validation could not be performed (Section 3.6). Domains follow manuscript Table 1: Demographic, Clinical, Severity, Laboratory, Device-support, Environmental. The ablation analysis fits three combined feature sets, in which the Clinical/Laboratory group holds the demographic, clinical, illness-severity and laboratory variables together.

Predictor	Domain	n missing	% missing
Platelets	Laboratory	4	2.07
Eosinophils	Laboratory	3	1.55
White blood cell count	Laboratory	3	1.55
Hematocrit	Laboratory	2	1.04
Hemoglobin	Laboratory	2	1.04
Lactate	Laboratory	2	1.04
Lymphocytes	Laboratory	2	1.04
Albumin	Laboratory	1	0.52
Creatinine	Laboratory	1	0.52
High FiO2 (> 40%)	Clinical	1	0.52
Bicarbonate	Laboratory	1	0.52
Neutrophils	Laboratory	1	0.52
Uric acid	Laboratory	1	0.52
ABO group	Clinical	0	0.00
APACHE II score	Severity	0	0.00
Admission diagnosis	Clinical	0	0.00
Age	Demographic	0	0.00
Blood urea nitrogen	Laboratory	0	0.00
C-reactive protein	Laboratory	0	0.00
Central venous catheter	Device-support	0	0.00
Cl	Laboratory	0	0.00
Comorbidity count	Clinical	0	0.00
Positive cultures	Clinical	0	0.00

Excessive visual	Environmental	0	0.00
Family visit	Environmental	0	0.00
Fever	Clinical	0	0.00
Glucose	Laboratory	0	0.00
High-flow oxygen	Device-support	0	0.00
Hospitalization within last 3 months	Clinical	0	0.00
Potassium	Laboratory	0	0.00
Invasive mechanical ventilation	Device-support	0	0.00
Malignancy	Clinical	0	0.00
Magnesium	Laboratory	0	0.00
Nasogastric tube	Device-support	0	0.00
Noninvasive mechanical ventilation	Device-support	0	0.00
Sodium	Laboratory	0	0.00
Newspaper	Environmental	0	0.00
Noise exposure	Environmental	0	0.00
PCT	Laboratory	0	0.00
Polypharmacy	Clinical	0	0.00
Renal replacement therapy	Device-support	0	0.00
Radio tablet	Environmental	0	0.00
Rh factor	Clinical	0	0.00
SOFA score	Severity	0	0.00
Female sex	Demographic	0	0.00
TV use	Environmental	0	0.00
Urinary cath	Device-support	0	0.00
Vasopressor use	Device-support	0	0.00
pCO2	Laboratory	0	0.00
pH	Laboratory	0	0.00

Table S2. Admission-diagnosis categories, frequencies and delirium rate (n = 193)

The admission-diagnosis variable was highly granular: 43 canonical categories, of which 25 occurred in a single patient. Four raw values were normalized before modelling - AC ÖDEMİ, DKA and YARA YERİ ENF were merged into the corresponding full-form categories, and the misspelling PANKERATİT was corrected to PANKREATİT - so that no clinical entity is split across categories. It was one-hot encoded with the encoder fitted on each training fold only (handle_unknown = 'ignore'); categories appearing solely in a held-out fold contributed no column. No rare-category collapsing was applied, because any collapsing rule derived from the full dataset would have reintroduced the leakage the pipeline is designed to prevent. The cost of this choice is that one-hot expansion raised the effective number of parameters to approximately 92, lowering the effective events-per-variable ratio below the nominal 1.96. Category labels were translated from the Turkish electronic medical record.

Admission diagnosis	N total	N delirium	N no-delirium	Delirium %
Acute kidney injury	41	25	16	61.0
Respiratory failure	34	20	14	58.8
Pneumonia	19	13	6	68.4
Gastrointestinal bleeding	15	4	11	26.7
COPD	10	5	5	50.0
Heart failure	7	3	4	42.9

Neutropenic fever	6	2	4	33.3
Liver failure	5	3	2	60.0
Pulmonary edema	5	2	3	40.0
Urosepsis	5	0	5	0.0
Diabetic ketoacidosis	4	0	4	0.0
Hypernatremia	3	3	0	100.0
Cholangiosepsis	3	1	2	33.3
Surgical-site infection	3	2	1	66.7
Alcohol intoxication	2	0	2	0.0
Decompensated heart failure	2	2	0	100.0
Chronic kidney disease	2	1	1	50.0
Type 2 respiratory failure	2	2	0	100.0
Acute kidney injury + pneumonia	1	1	0	100.0
Acute coronary syndrome	1	0	1	0.0
Anemia	1	0	1	0.0
ARDS	1	1	0	100.0
Cerebral edema	1	1	0	100.0
Carbon monoxide intoxication	1	0	1	0.0
Electrolyte disturbance	1	0	1	0.0
Chronic kidney disease + pulmonary edema	1	0	1	0.0
COPD exacerbation	1	0	1	0.0
Cholangitis	1	1	0	100.0
Cholestasis	1	0	1	0.0
Chronic renal failure	1	1	0	100.0
Chronic liver failure	1	1	0	100.0
Meningitis	1	1	0	100.0
Multiple myeloma	1	1	0	100.0
Pancreatitis	1	0	1	0.0
Peritonitis	1	0	1	0.0
Post-cardiac-arrest state	1	0	1	0.0
Pulmonary embolism	1	0	1	0.0
Rhabdomyolysis	1	0	1	0.0
Sepsis	1	1	0	100.0
Central nervous system infection	1	0	1	0.0
Ileus	1	1	0	100.0
Infective endocarditis	1	0	1	0.0
Ischemic hepatitis	1	0	1	0.0

Table S3. Complete baseline comparison for all 50 candidate predictors

Manuscript Table 2 shows only the predictors reaching p < 0.05. The complete comparison of all 50 candidate predictors between the delirium and no-delirium groups is given below: median [IQR] for continuous and ordinal variables, n (%) for binary variables, with the test used, the raw p-value and the Benjamini-Hochberg FDR-adjusted q-value. Fifteen predictors reached raw p < 0.05 and 11 survived FDR correction (q < 0.05). The four that reached raw significance but did not survive correction were age (q = 0.052), sex (q = 0.080), blood urea nitrogen (q = 0.080) and comorbidity count (q = 0.128). Domains (Demographic, Clinical, Severity, Laboratory, Device-support, Environmental) and measurement types follow manuscript Table 1; the test column records how each variable was compared, which is not the same thing - ordinal variables were compared with the Mann-Whitney U test. The admission-diagnosis distribution is given separately in Table S2.

Predictor	Domain	Type	Overall (n = 193)	Delirium (n = 98)	No delirium (n = 95)	Test	p	q (BH)	Significance
Age	Demographic	Continuous	69.0 [56.0–79.0]	70.0 [60.0–80.0]	66.0 [49.0–77.0]	Mann–Whitney U = 5626	0.012	0.052	p < 0.05 only
Female sex	Demographic	Binary	89 (46.1%)	37 (37.8%)	52 (54.7%)	Fisher exact	0.021	0.080	p < 0.05 only
Admission diagnosis	Clinical	Categorical	see Table S2	see Table S2	see Table S2	χ^2 = 53.72	0.106	0.312	ns
Fever	Clinical	Binary	27 (14.0%)	12 (12.2%)	15 (15.8%)	Fisher exact	0.537	0.746	ns
Vasopressor use	Device-support	Binary	60 (31.1%)	47 (48.0%)	13 (13.7%)	Fisher exact	< 0.001	< 0.001	q < 0.05
Hospitalization within last 3 months	Clinical	Binary	109 (56.5%)	67 (68.4%)	42 (44.2%)	Fisher exact	< 0.001	0.013	q < 0.05
High FiO2 (> 40%)	Clinical	Binary	79 (40.9%)	51 (52%)	28 (29.5%)	Fisher exact	0.001	0.013	q < 0.05
Invasive mechanical ventilation	Device-support	Ordinal	0.0 [0.0–0.0]	0.0 [0.0–1.0]	0.0 [0.0–0.0]	Mann–Whitney U = 5432	0.007	0.034	q < 0.05
Noninvasive mechanical ventilation	Device-support	Binary	86 (44.6%)	54 (55.1%)	32 (33.7%)	Fisher exact	0.004	0.021	q < 0.05
High-flow oxygen	Device-support	Binary	63 (32.6%)	42 (42.9%)	21 (22.1%)	Fisher exact	0.002	0.016	q < 0.05
Renal replacement therapy	Device-support	Binary	56 (29.0%)	29 (29.6%)	27 (28.4%)	Fisher exact	0.875	1.000	ns
Central venous catheter	Device-support	Binary	98 (50.8%)	55 (56.1%)	43 (45.3%)	Fisher exact	0.151	0.324	ns
Nasogastric tube	Device-support	Ordinal	0.0 [0.0–1.0]	1.0 [0.0–1.0]	0.0 [0.0–0.0]	Mann–Whitney U = 6367	< 0.001	< 0.001	q < 0.05
Urinary cath	Device-support	Binary	174 (90.2%)	92 (93.9%)	82 (86.3%)	Fisher exact	0.093	0.292	ns
Polypharmacy	Clinical	Binary	69 (35.8%)	37 (37.8%)	32 (33.7%)	Fisher exact	0.652	0.836	ns
Family visit	Environmental	Binary	178 (92.2%)	92 (93.9%)	86 (90.5%)	Fisher exact	0.430	0.669	ns
Noise exposure	Environmental	Binary	34 (17.6%)	17 (17.3%)	17 (17.9%)	Fisher exact	1.000	1.000	ns
Radio tablet	Environmental	Binary	43 (22.3%)	17 (17.3%)	26 (27.4%)	Fisher exact	0.119	0.314	ns
TV use	Environmental	Binary	6 (3.1%)	3 (3.1%)	3 (3.2%)	Fisher exact	1.000	1.000	ns
Newspaper	Environmental	Binary	2 (1.0%)	1 (1.0%)	1 (1.1%)	Fisher exact	1.000	1.000	ns
Excessive visual	Environmental	Binary	8 (4.1%)	3 (3.1%)	5 (5.3%)	Fisher exact	0.493	0.704	ns
Comorbidity count	Clinical	Ordinal	2.0 [1.0–2.0]	2.0 [1.0–2.0]	2.0 [1.0–2.0]	Mann–Whitney U = 5360	0.038	0.128	p < 0.05 only
Malignancy	Clinical	Binary	49 (25.4%)	25 (25.5%)	24 (25.3%)	Fisher exact	1.000	1.000	ns
Glucose	Laboratory	Continuous	118.0 [93.0–161.0]	116.5 [92.0–156.0]	118.0 [94.0–162.0]	Mann–Whitney U = 4513	0.715	0.872	ns
ABO group	Clinical	Ordinal	1.0 [0.0–1.0]	1.0 [0.0–1.0]	1.0 [0.0–1.5]	Mann–Whitney U = 4680	0.946	1.000	ns
Rh factor	Clinical	Binary	166 (86.0%)	84 (85.7%)	82 (86.3%)	Fisher exact	1.000	1.000	ns
Positive cultures	Clinical	Binary	77 (39.9%)	40 (40.8%)	37 (38.9%)	Fisher exact	0.883	1.000	ns
SOFA score	Severity	Ordinal	5.0 [4.0–7.0]	6.0 [4.0–7.0]	4.0 [3.0–6.0]	Mann–Whitney U = 5801	0.003	0.018	q < 0.05
APACHE II score	Severity	Ordinal	20.0 [16.0–25.0]	22.0 [17.0–25.0]	18.0 [14.5–25.0]	Mann–Whitney U = 5693	0.007	0.034	q < 0.05
pH	Laboratory	Continuous	7.4 [7.3–7.4]	7.4 [7.3–7.4]	7.4 [7.3–7.4]	Mann–Whitney U = 4232	0.276	0.475	ns

pCO2	Laboratory	Continuous	36.0 [31.0–44.0]	37.0 [31.0–44.8]	35.0 [30.5–44.0]	Mann–Whitney U = 4958	0.435	0.669	ns
Bicarbonate	Laboratory	Continuous	24.0 [19.8–28.0]	23.0 [20.5–29.0]	24.0 [19.0–28.0]	Mann–Whitney U = 4958	0.363	0.605	ns
Lactate	Laboratory	Continuous	2.1 [1.5–3.3]	2.3 [1.6–3.4]	1.9 [1.5–3.1]	Mann–Whitney U = 5114	0.146	0.324	ns
White blood cell count	Laboratory	Continuous	10.8 [7.5–15.3]	11.0 [8.0–15.4]	10.2 [7.0–15.0]	Mann–Whitney U = 4778	0.480	0.704	ns
Hemoglobin	Laboratory	Continuous	9.1 [7.8–11.1]	9.0 [7.9–10.6]	9.2 [7.8–12.3]	Mann–Whitney U = 3955	0.114	0.314	ns
Hematocrit	Laboratory	Continuous	28.7 [22.8–35.7]	28.3 [23.6–32.7]	29.9 [22.1–36.9]	Mann–Whitney U = 4135	0.268	0.475	ns
Platelets	Laboratory	Continuous	175.0 [61.0–258.0]	172.0 [55.2–271.5]	175.0 [63.0–249.0]	Mann–Whitney U = 4589	0.741	0.881	ns
Neutrophils	Laboratory	Continuous	8.6 [5.8–13.0]	9.1 [6.0–13.2]	7.8 [5.0–12.6]	Mann–Whitney U = 5178	0.139	0.324	ns
Lymphocytes	Laboratory	Continuous	0.9 [0.5–1.7]	0.9 [0.5–1.6]	0.9 [0.5–1.9]	Mann–Whitney U = 4407	0.695	0.869	ns
Eosinophils	Laboratory	Continuous	0.0 [0.0–0.1]	0.0 [0.0–0.1]	0.0 [0.0–0.1]	Mann–Whitney U = 4696	0.618	0.813	ns
Blood urea nitrogen	Laboratory	Continuous	78.0 [47.0–123.0]	89.0 [54.5–133.0]	70.0 [38.5–111.0]	Mann–Whitney U = 5542	0.022	0.080	p < 0.05 only
Creatinine	Laboratory	Continuous	1.4 [0.9–3.0]	1.7 [1.0–3.1]	1.3 [0.8–2.8]	Mann–Whitney U = 5198	0.126	0.314	ns
Sodium	Laboratory	Continuous	137.0 [134.0–140.0]	136.0 [133.0–139.0]	137.0 [134.0–140.0]	Mann–Whitney U = 4144	0.188	0.365	ns
Cl	Laboratory	Continuous	102.0 [98.0–107.0]	102.0 [98.0–107.0]	102.0 [97.0–107.0]	Mann–Whitney U = 4872	0.577	0.780	ns
Potassium	Laboratory	Continuous	4.1 [3.6–4.9]	4.1 [3.7–5.0]	4.0 [3.5–4.8]	Mann–Whitney U = 5164	0.190	0.365	ns
Magnesium	Laboratory	Continuous	1.9 [1.7–2.2]	1.9 [1.7–2.3]	1.9 [1.7–2.2]	Mann–Whitney U = 5123	0.227	0.420	ns
C-reactive protein	Laboratory	Continuous	73.0 [33.0–176.0]	95.0 [51.2–189.2]	54.0 [19.5–138.5]	Mann–Whitney U = 5880	0.002	0.013	q < 0.05
PCT	Laboratory	Continuous	0.7 [0.2–3.7]	0.6 [0.2–2.8]	0.8 [0.2–4.8]	Mann–Whitney U = 4356	0.442	0.669	ns
Albumin	Laboratory	Continuous	30.0 [25.5–34.2]	28.5 [25.0–32.5]	32.0 [27.4–36.0]	Mann–Whitney U = 3338	0.001	0.013	q < 0.05
Uric acid	Laboratory	Continuous	6.1 [4.4–8.6]	6.8 [4.6–9.0]	5.8 [4.1–8.1]	Mann–Whitney U = 5153	0.156	0.324	ns

Table S4. Extended comparison with previously published ICU delirium prediction models

This is the extended version of manuscript Table 10. It carries the same seven models and adds the predictor count for each, which Table 10 omits for space; the other columns match Table 10. Published models can lose discrimination on external validation (PRIDE 0.916 → 0.721; Zhang et al. 0.793 → 0.701). The present estimates—0.696 for the full model, 0.756 for the fixed exploratory reduced model, and 0.700 with nested feature selection—are internal estimates and should not be interpreted as external performance. External validation remains necessary.

Model (ref.)	Cohort / setting	n	Predictors	Validation	Model type	AUROC (development)	AUROC (external)
PRE-DELIRIC [16]	Dutch multicenter ICU	1,613	10 (24 h)	Development + external	Logistic regression	0.87	0.71-0.77 [17,18]
E-PRE-DELIRIC [20]	Multinational ICU	~2,000	9 (admission)	Multinational	Logistic regression	0.76	-
PRIDE [21]	Korean tertiary ICU	large EHR	structured EHR	Internal + external	Machine learning	0.916	0.721
Zhang et al. [29]	MIMIC-IV / eICU-CRD	large	structured EHR	Internal + external	XGBoost	0.793	0.701
Wang et al. [30]	Taiwanese ICU	large	admission data	Development + deployment	Machine learning	high internal	-
Turkish PRE-DELIRIC validation [23]	Turkish ICU	172	10	External validation	Logistic regression (applied)	-	0.649

This study	Turkish internal-medicine ICU	193	50 (full) / 11 (reduced)	Internal (nested 5×4 CV, leakage-aware)	Elastic-net logistic regression	0.696 (full); 0.756 reduced (exploratory); 0.700 nested selection	not yet performed

Table S5. Full coefficient table and mathematical form of the fitted elastic-net logistic regression model

Log-odds coefficients and odds ratios with 95% bootstrap confidence intervals (500 resamples; seed = 42) for all 92 encoded features at the modal penalty (l1_ratio = 0.5, C = 0.05), ordered by absolute coefficient. The admission-diagnosis one-hot columns are included for completeness; the L1 component shrank all of them to exactly OR = 1.000. Only vasopressor use and nasogastric tube have a confidence interval that strictly excludes OR = 1.000. Domains follow manuscript Table 1: Demographic, Clinical, Severity, Laboratory, Device-support, Environmental. The ablation analysis fits three combined feature sets, in which the Clinical/Laboratory group holds the demographic, clinical, illness-severity and laboratory variables together.

Encoded feature	Domain	Coefficient (log-odds)	OR	95% bootstrap CI	CI strictly excludes OR = 1.000
Vasopressor use	Device-support	0.335	1.398	1.089-1.747	yes
Nasogastric tube	Device-support	0.326	1.386	1.066-1.696	yes
Albumin	Laboratory	-0.135	0.873	0.689-1.000	no
High-flow oxygen	Device-support	0.105	1.111	1.000-1.377	no
Hospitalization within last 3 months	Clinical	0.088	1.092	1.000-1.358	no
APACHE II score	Severity	0.043	1.044	1.000-1.255	no
Age	Demographic	0.037	1.038	1.000-1.258	no
High FiO2 (> 40%)	Clinical	0.035	1.035	1.000-1.263	no
Noninvasive mechanical ventilation	Device-support	0.032	1.032	1.000-1.265	no
Female sex	Demographic	-0.030	0.970	0.753-1.000	no
Admission diagnosis: Heart failure	Clinical	0.000	1.000	1.000-1.000	no
Admission diagnosis: Cholangitis	Clinical	0.000	1.000	1.000-1.000	no
Admission diagnosis: Cholangiosepsis	Clinical	0.000	1.000	1.000-1.000	no
Admission diagnosis: COPD exacerbation	Clinical	0.000	1.000	1.000-1.000	no
Admission diagnosis: COPD	Clinical	0.000	1.000	1.000-1.000	no
Admission diagnosis: Chronic kidney disease + pulmonary edema	Clinical	0.000	1.000	1.000-1.000	no
Admission diagnosis: Chronic kidney disease	Clinical	0.000	1.000	1.000-1.000	no
Admission diagnosis: Liver failure	Clinical	0.000	1.000	1.000-1.000	no
Admission diagnosis: Gastrointestinal bleeding	Clinical	0.000	1.000	1.000-1.000	no
Admission diagnosis: Hypernatremia	Clinical	0.000	1.000	1.000-1.000	no
Admission diagnosis: Electrolyte disturbance	Clinical	0.000	1.000	1.000-1.000	no
Admission diagnosis: Diabetic ketoacidosis	Clinical	0.000	1.000	1.000-1.000	no
Admission diagnosis: Cholestasis	Clinical	0.000	1.000	1.000-1.000	no
Admission diagnosis: Carbon monoxide intoxication	Clinical	0.000	1.000	1.000-1.000	no
Admission diagnosis: Cerebral edema	Clinical	0.000	1.000	1.000-1.000	no
Admission diagnosis: Anemia	Clinical	0.000	1.000	1.000-1.000	no
Admission diagnosis: ARDS	Clinical	0.000	1.000	1.000-1.000	no
Admission diagnosis: Decompensated heart failure	Clinical	0.000	1.000	1.000-1.000	no
Admission diagnosis: Meningitis	Clinical	0.000	1.000	1.000-1.000	no
Admission diagnosis: Chronic renal failure	Clinical	0.000	1.000	1.000-1.000	no
Admission diagnosis: Rhabdomyolysis	Clinical	0.000	1.000	1.000-1.000	no
Admission diagnosis: Infective endocarditis	Clinical	0.000	1.000	1.000-1.000	no
Admission diagnosis: Ileus	Clinical	0.000	1.000	1.000-1.000	no
Admission diagnosis: Urosepsis	Clinical	0.000	1.000	1.000-1.000	no
Admission diagnosis: Surgical-site infection	Clinical	0.000	1.000	1.000-1.000	no
Admission diagnosis: Type 2 respiratory failure	Clinical	0.000	1.000	1.000-1.000	no

Admission diagnosis: Central nervous system infection	Clinical	0.000	1.000	1.000-1.000	no
Admission diagnosis: Respiratory failure	Clinical	0.000	1.000	1.000-1.000	no
Admission diagnosis: Sepsis	Clinical	0.000	1.000	1.000-1.000	no
Admission diagnosis: Pulmonary edema	Clinical	0.000	1.000	1.000-1.000	no
Admission diagnosis: Chronic liver failure	Clinical	0.000	1.000	1.000-1.000	no
Admission diagnosis: Pulmonary embolism	Clinical	0.000	1.000	1.000-1.000	no
Admission diagnosis: Post-cardiac-arrest state	Clinical	0.000	1.000	1.000-1.000	no
Admission diagnosis: Pneumonia	Clinical	0.000	1.000	1.000-1.000	no
Admission diagnosis: Peritonitis	Clinical	0.000	1.000	1.000-1.000	no
Admission diagnosis: Pancreatitis	Clinical	0.000	1.000	1.000-1.000	no
Admission diagnosis: Neutropenic fever	Clinical	0.000	1.000	1.000-1.000	no
Admission diagnosis: Multiple myeloma	Clinical	0.000	1.000	1.000-1.000	no
Admission diagnosis: Acute coronary syndrome	Clinical	0.000	1.000	1.000-1.000	no
Admission diagnosis: Alcohol intoxication	Clinical	0.000	1.000	1.000-1.000	no
PCT	Laboratory	0.000	1.000	0.923-1.000	no
Admission diagnosis: Acute kidney injury + pneumonia	Clinical	0.000	1.000	1.000-1.000	no
SOFA score	Severity	0.000	1.000	1.000-1.180	no
Rh factor	Clinical	0.000	1.000	0.946-1.040	no
ABO group	Clinical	0.000	1.000	1.000-1.053	no
Glucose	Laboratory	0.000	1.000	1.000-1.035	no
Malignancy	Clinical	0.000	1.000	0.953-1.000	no
Comorbidity count	Clinical	0.000	1.000	1.000-1.194	no
Excessive visual	Environmental	0.000	1.000	0.999-1.000	no
Newspaper	Environmental	0.000	1.000	1.000-1.000	no
TV use	Environmental	0.000	1.000	0.968-1.075	no
Radio tablet	Environmental	0.000	1.000	0.887-1.000	no
Noise exposure	Environmental	0.000	1.000	0.938-1.000	no
Family visit	Environmental	0.000	1.000	1.000-1.191	no
Polypharmacy	Clinical	0.000	1.000	1.000-1.028	no
Urinary cath	Device-support	0.000	1.000	1.000-1.125	no
Central venous catheter	Device-support	0.000	1.000	1.000-1.059	no
Renal replacement therapy	Device-support	0.000	1.000	0.949-1.000	no
Invasive mechanical ventilation	Device-support	0.000	1.000	1.000-1.063	no
Fever	Clinical	0.000	1.000	0.864-1.000	no
Positive cultures	Clinical	0.000	1.000	0.865-1.000	no
pH	Laboratory	0.000	1.000	0.955-1.000	no
Admission diagnosis: Acute kidney injury	Clinical	0.000	1.000	1.000-1.000	no
pCO2	Laboratory	0.000	1.000	1.000-1.019	no
Uric acid	Laboratory	0.000	1.000	1.000-1.176	no
C-reactive protein	Laboratory	0.000	1.000	1.000-1.196	no
Magnesium	Laboratory	0.000	1.000	1.000-1.044	no
Potassium	Laboratory	0.000	1.000	1.000-1.033	no
Cl	Laboratory	0.000	1.000	1.000-1.206	no
Sodium	Laboratory	0.000	1.000	0.945-1.000	no
Creatinine	Laboratory	0.000	1.000	1.000-1.022	no
Blood urea nitrogen	Laboratory	0.000	1.000	1.000-1.058	no
Eosinophils	Laboratory	0.000	1.000	0.887-1.000	no
Lymphocytes	Laboratory	0.000	1.000	1.000-1.000	no
Neutrophils	Laboratory	0.000	1.000	0.979-1.000	no
Platelets	Laboratory	0.000	1.000	1.000-1.033	no

Hematocrit	Laboratory	0.000	1.000	0.965-1.000	no
Hemoglobin	Laboratory	0.000	1.000	0.976-1.000	no
White blood cell count	Laboratory	0.000	1.000	1.000-1.000	no
Lactate	Laboratory	0.000	1.000	1.000-1.083	no
Bicarbonate	Laboratory	0.000	1.000	1.000-1.075	no
Admission diagnosis: Ischemic hepatitis	Clinical	0.000	1.000	1.000-1.000	no

Mathematical model form. The fitted model is a linear logistic function

$logit(P[delirium]) = \beta_0 + \Sigma\ \beta_j \cdot z_j$

where z_j are the standardized (or one-hot encoded) predictors and β_j the elastic-net coefficients at the modal penalty (l1_ratio = 0.5, C = 0.05). The coefficients are listed here for transparency and reproducibility of the reported analysis. They are not a deployable clinical equation. The intercept, the fold-specific standardization means and standard deviations, the imputation values and the isotonic calibration mapping are not reproduced here, so the coefficients alone cannot generate a calibrated probability for an individual patient. The model is internally validated only, its predicted probabilities require recalibration, and it should not be applied to individual patients without external validation.

Table S6. Classification metrics and confusion matrix at each model's Youden-optimal threshold (24-point GBM comparator)

Counts are computed on the pooled out-of-fold predictions (n = 98 delirium, 95 no-delirium) at each model's Youden-optimal threshold. All rates are derived from the counts in the same row. Threshold-dependent rates for the full model and the gradient boosting comparator are also given in manuscript Table 4.

Model	Threshold	TP	FN	TN	FP	Sensitivity	Specificity	PPV	NPV	F1	Accuracy
Regularized LR (full, 50 features)	0.497	70	28	61	34	71.4%	64.2%	67.3%	68.5%	0.693	67.9%
GBM comparator (24-point grid)	0.506	70	28	59	36	71.4%	62.1%	66.0%	67.8%	0.686	66.8%
Regularized LR (fixed reduced, 11 features)	0.581	61	37	75	20	62.2%	78.9%	75.3%	67.0%	0.682	70.5%

Table S7. Model variants and sensitivity analyses

All analyses use the identical 5-fold outer × 4-fold inner nested cross-validation protocol and pooled out-of-fold predictions. The second row is the analysis requested by the reviewers: repeating the entire bootstrap feature-selection procedure inside each outer training fold removes the reduced model's apparent advantage, which identifies that advantage as selection-induced optimism. Bootstrap intervals for the Hosmer-Lemeshow p-value are not reported: at n = 193 they span almost the whole unit interval and would invite over-interpretation.

Analysis	Result	Interpretation
Reduced model, selection on the full dataset (exploratory)	AUROC 0.756 (95% CI: 0.684–0.819)	Optimistic: predictors were selected on the evaluation data
Reduced model, feature selection nested within each fold	AUROC 0.700 (95% CI: 0.623–0.773)	Equivalent to the full model (DeLong p = 0.87); significantly below the fixed reduced model (p = 0.007)
Predictors selected in all five folds	Albumin, nasogastric tube, vasopressor use	Only 3 of 11 stable (6–9 predictors selected per fold), indicating selection instability under low EPV
Untuned baseline (no grid search)	AUROC 0.627 (95% CI: 0.544–0.710)	Reference point only; the baseline also differs in penalty type (L2 at C = 1.0) and lacks isotonic

		calibration, so the gap cannot be attributed to hyperparameter tuning alone
Reduced model without SOFA and APACHE II	AUROC 0.764 (95% CI: 0.695–0.827)	No loss relative to 0.756 (DeLong p = 0.44); the composite severity scores add no discrimination beyond the individual predictors
Brier score, reduced − full	−0.023 (95% CI: −0.039 to −0.004); p = 0.02	Lower apparent score; exploratory and affected by fixed feature selection
Full-model calibration slope	0.741 (95% CI: 0.345–1.265)	CI includes 1.0, so no departure from a slope of 1 is detectable at this sample size
Reduced-model calibration slope	0.235 (95% CI: 0.068–0.886)	CI excludes 1.0, supporting the conclusion that the probabilities are too extreme; uncertainty remains
